# Computer-Aided Estimation of Biological Activity Profiles of Drug-Like Compounds Taking into Account Their Metabolism in Human Body

**DOI:** 10.3390/ijms21207492

**Published:** 2020-10-11

**Authors:** Dmitry A. Filimonov, Anastassia V. Rudik, Alexander V. Dmitriev, Vladimir V. Poroikov

**Affiliations:** Laboratory of Structure-Function Based Drug Design, Department of Bioinformatics, Institute of Biomedical Chemistry, Pogodinskaya Str., 10, bldg. 8, 119121 Moscow, Russia; dmitry.filimonov@ibmc.msk.ru (D.A.F.); rudik_anastassia@mail.ru (A.V.R.); a.v.dmitriev@mail.ru (A.V.D.)

**Keywords:** drug-like compounds, biological activity profiles, metabolism, computer-aided predictions

## Abstract

Most pharmaceutical substances interact with several or even many molecular targets in the organism, determining the complex profiles of their biological activity. Moreover, due to biotransformation in the human body, they form one or several metabolites with different biological activity profiles. Therefore, the development and rational use of novel drugs requires the analysis of their biological activity profiles, taking into account metabolism in the human body. In silico methods are currently widely used for estimating new drug-like compounds’ interactions with pharmacological targets and predicting their metabolic transformations. In this study, we consider the estimation of the biological activity profiles of organic compounds, taking into account the action of both the parent molecule and its metabolites in the human body. We used an external dataset that consists of 864 parent compounds with known metabolites. It is shown that the complex assessment of active pharmaceutical ingredients’ interactions with the human organism increases the quality of computer-aided estimates. The toxic and adverse effects showed the most significant difference: reaching 0.16 for recall and 0.14 for precision.

## 1. Introduction

The discovery and development of new drugs is a topical biomedical research task due to the unmet needs in treating many human disorders and insufficient efficacy and safety of the launched medicines [1]. Currently, the “magic bullet” concept proposed by the Nobel laureate Paul Ehrlich at the beginning of the XX century [2] is substituted by the network pharmacology approach [3]. Such changes were essential because most pharmaceutical agents interact with multiple molecular targets in the organism, producing desirable and adverse pharmacological effects. Pleiotropic action of pharmaceuticals is caused by both the parent compound and its metabolites that arise during the biotransformation of drug substances in the human body [4]. Due to the unsuspected toxicity of drug metabolites, many launched pharmaceuticals have been withdrawn from the market; some examples include troglitazone, tienilic acid, ximelagatran, zomepirac, etc. For instance, troglitazone was approved for medical purposes as an antidiabetic and anti-inflammatory drug in 1997 and withdrawn in 2000 due to the hepatotoxicity mainly produced by its reactive metabolites [5].

Detailed consideration of the drug’s effects on the organism must include the complex analysis of chemical–biological interactions, which may be performed using up-to-date chemoinformatics approaches [6]. Machine learning methods allow for estimating drug–target interactions with reasonable accuracy, which produces probable biological activity profiles [7,8,9,10] and different characteristics of drug metabolism [4,11,12,13]. However, to the best of our knowledge, an investigation that combine the in silico estimates of drug metabolism and predictions of the biological activity profiles for both the parent drug and its metabolites, has never been conducted. The purpose of our ongoing studies is the development of the chemoinformatics method for the assessment of biological activity profiles of drug-like compounds, taking into account their metabolism in the human body. In this article, we present the evaluation of biological activity predictions obtained with the use of a computer program PASS (Prediction of Activity Spectra for Substances) [7,14,15] and experimentally determined metabolic transformations of drug substances presented in two publicly available databases DrugBank [16] and ChEMBL [17]. The results clearly demonstrate that integration of in silico predicted biological activity profiles of the parent drug substance and its metabolites improves the computer-aided assessments.

## 2. Results

### 2.1. Information about Drug Substances and Their Metabolites

We extracted the information about structural formulae of pharmaceutical substances and their metabolites from DrugBank version 5.1.6 (822 substances) and ChEMBL version 26 (142 substances). The total number of parent compounds and their metabolites equals to 4249. The data presented in the field “Drug categories” in DrugBank and "Max_phase" in ChEMBL shows that about 30% of the selected structures belong to the “investigational drugs”; thus, about 70% of structures represent the launched drugs. The complete list of the analyzed pharmaceutical substances is provided in Supplementary Materials (Table S1). 

**Figure 1:**
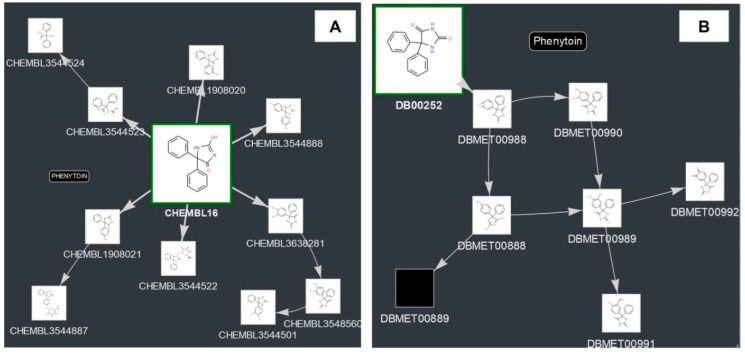
Metabolism graphs for Phenytoin: (**A**) ChEMBL (http://www.way2drug.com/metapass/view_path_new.php?id=93). (**B**) DrugBank (http://www.way2drug.com/metapass/view_path_new.php?id=197).

Using the information about the experimentally determined biotransformation schemes from DrugBank and ChEMBL, we obtained the metabolism graphs for each pharmaceutical substance. Figure 1A shows an example of the metabolism graph for Phenytoin based on the information from ChEMBL. The directed graph represents the nodes (chemical compounds) and the edges between them (the direction is related to the transformation). Each node is represented by an image of the structural formula and the identifier of the corresponding database. Cases where there is no image for the compound (for example, DBMET00889 for Phenytoin) means that the original data source (ChEMBL or DrugBank) did not have any structure for it. The node associated with the parent compound is larger and highlighted in green.

Sometimes, the biotransformation schemes presented in ChEMBL and DrugBank differ (Figure 1A,B). DrugBank contains information about seven metabolites of Phenytoin produced by aromatic hydroxylation, C-oxidation, and epoxidation reactions. In ChEMBL, three additional metabolites produced by Glucuronidation, O-Methylation, Ring-opening, and Hydrolysis are also presented. The number of different biotransformation schemes in ChEMBL and DrugBank is 964. Thus, in this study we decided to analyze both metabolism graphs as the reasons for the unambiguous selection of a particular biotransformation scheme either from DrugBank or from ChEMBL are not evident. 

**Figure 2:**
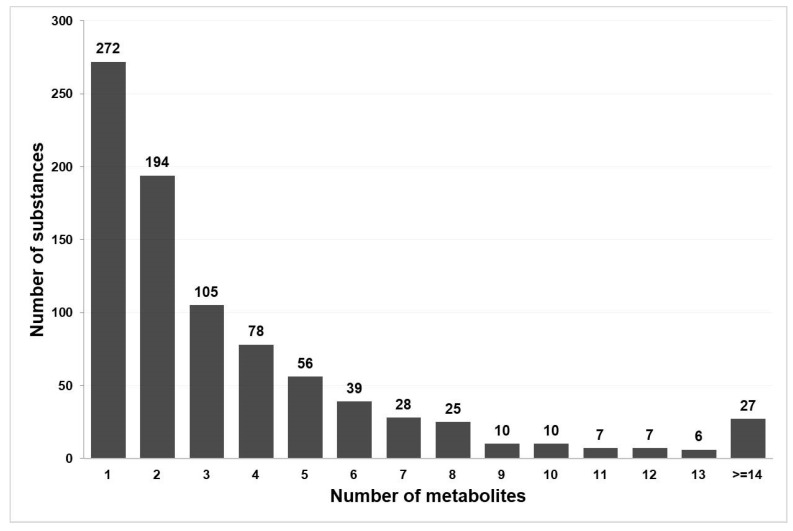
The distribution of the analyzed drug substances by the number of metabolites.

The total number of substances with a known metabolism graph is 880. However, in some cases, data extracted from DrugBank and ChEMBL does not have information about metabolites’ structural formulae. After excluding such records, we obtained 864 metabolism graphs for 782 drug substances, which (1) contain the structural formulae of metabolites and (2) are included in the PASS training set. The distribution of substances by the number of known metabolites is shown in Figure 2. As one may see, over 30% of metabolism graphs contain information about only one metabolite.

### 2.2. Accuracy of Prediction of Biological Activity of Compounds with and without Considering their Metabolites

Since in preclinical studies, the biological activities are determined in vivo using experimental animals, both parent and metabolites may cause the detected pharmacological action. In clinical trials, the observed pharmacotherapeutic and adverse effects are investigated in patients; therefore, the integrated response on the parent substance and its metabolites is also responsible for the administered drug’s observed actions.

Using PASS (see Section 4), we compared the accuracy of prediction of biological activity for a substance, taking into account (1) only the parent compound and (2) the parent compound and all its metabolites. In the first case, we consider the Pa value of the probability of belonging to the class of “actives” as the criteria to estimate the predicted activity spectra for each of the 864 drug substances. In the second case, for each activity, the Pa_max value was determined as the maximum of Pa values calculated for the parent compound and all its metabolites. Pa_max values were used as the criteria to estimate the predicted activity spectra for the analyzed drug substances. In both cases, the predicted activity spectra were compared with the known activity spectra included in the PASS training set.

We supposed that the compound has a particular activity if the Pa or Pa_max values that exceeded the particular threshold varied from 0.1 to 0.9. We have considered four categories of biological activity: pharmacological effects, mechanisms of action, antitargets, toxic and adverse effects. The lists of biological activities belonging to those categories are given in the Supplementary Materials (Tables S2–S5). 

**Figure 3:**
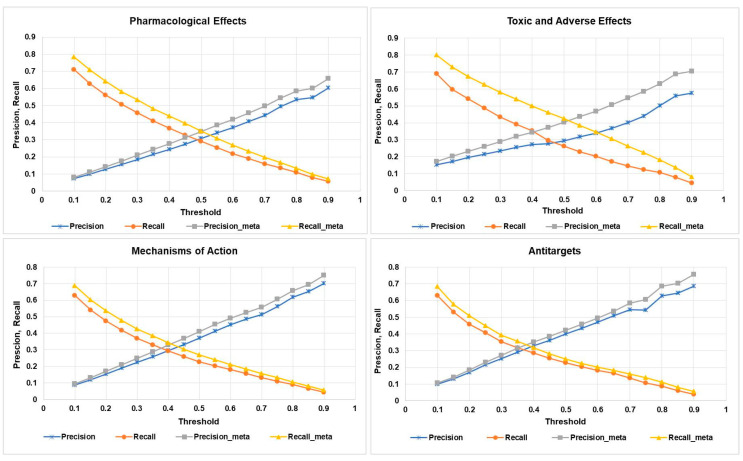
Average precision and recall estimates for different categories of biological activities (X axis represents the threshold values).

In this study, we used the “Precision” and “Recall” values as the (Q)SAR quality metrics (see Section 4.4). The obtained average quality metrics values are shown in Figure 3. Precision and recall values are calculated with the use of the predicted activity spectra only for the parent compound; precision-meta and recall-meta values are calculated using the predicted activity spectra for the parent compound and all its metabolites.

As one may see from Figure 3, for all categories of activity at any threshold, correspondence between the predicted and known biological activity spectra is better if we considered the effect of the metabolites. The most substantial difference is observed for the toxic and adverse effects, which at the threshold value 0.5 achieves 0.16 for recall, and at the threshold value 0.7 achieves 0.14 for precision metrics. The result demonstrates that considering the metabolism of the parent drug substance, increases the chances to identify the potentially toxic metabolites, based on computational predictions (see the particular examples in Section 2.3).

From Figure 3, it is also clear that the best balance between the recall and precision is achieved if the Pa value corresponds to the range 0.4 to 0.5. 

**Figure 4:**
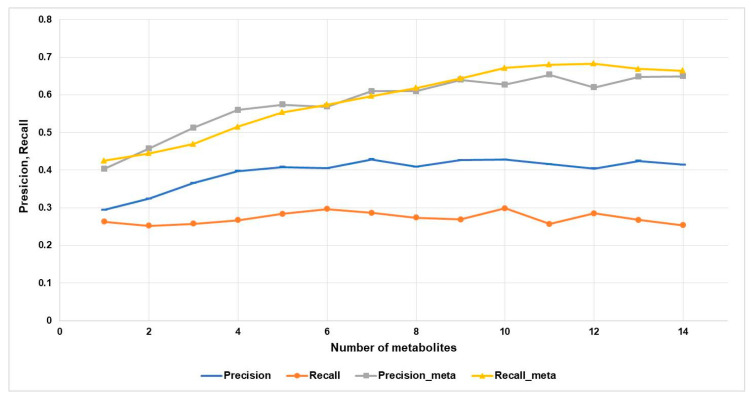
Accuracy metrics estimates depending on the number of metabolites for toxic and adverse Effects category at the 0.5 threshold.

We analyzed how quality metrics depend on the number of metabolites (*n*) for the toxic and adverse effects category at Pa > 0.5 or (Pa_max > 0.5). Figure 4 shows the precision, recall, precision-meta, and recall-meta quality metrics depending on *n*.

According to Figure 4, the precision value is increased from ~0.3 to ~0.4. The precision-meta and recall-meta values are increased from ~0.4 to ~0.65 if the biological activity spectra of more metabolites are taken into account. On the contrary, the recall values do not depend on the number of known metabolites and are almost the same for the only metabolite and fourteen ones.

### 2.3. Examples of Predictions for Some Drugs

#### 2.3.1. Toxic and Adverse Effects Predicted for Phenytoin and Its Metabolites

In 1939, Pfizer launched the voltage-gated sodium channel blocker Phenytoin for the treatment of epilepsy and arrhythmia [18]. Due to the moderate cost of therapy, it was included in the World Health Organization’s List of Essential Medicines and became one of the most widely used anticonvulsants. However, Phenytoin has a relatively narrow therapeutic index that may become the reason for a wide variety of adverse and toxic effects [19], particularly in overdose. 

As it has been already mentioned in Section 2.1, the metabolism graphs of Phenytoin presented in ChEMBL and DrugBank differ one from another. Therefore, we consider how the analysis of the estimated biological activity profiles for the parent compound and its metabolites influence the accuracy of predictions for toxic and adverse effects in both cases.

The integration of the prediction results of adverse and toxic effects for Phenytoin and its metabolites is shown in Table 1 (metabolism graph from ChEMBL).

The table contains only activities that were predicted with Pa > Pi for Phenytoin (Pa is the probability of belonging to the class of “actives” and Pi is the probability of belonging to the class of “inactives”). For all predicted activities Pa_max > Pa. Known activities are highlighted in bold.

As one may see from the data presented in Table 1, the probabilities of all known activities calculated for some metabolites exceeded those calculated for the parent compound. In particular, Pa values for teratogenic activity increased from 0.534 to 0.844; for carcinogenic activity—from 0.571 to 0.831; for embryotoxic activity—from 0.495 to 0.829; etc. 

Similar results were obtained when the metabolism graph for Phenytoin from DrugBank was analyzed. For example, Pa estimations for carcinogenic and embryotoxic activities for the parent compound equal to 0.362 and 0.476, while for its metabolite DBMET00990 they equal to 0.743 and 0.647, respectively. 

It was found that Phenytoin’s reactive metabolites cause drug-induced liver injury [20]. Among the adverse drug effects [21] predicted for Phenytoin and its metabolites (Supplementary Materials, Table S6), hepatotoxicity of the parent structure is predicted with Pa = 0.782, but Pa = 0.876 for its metabolite CHEMBL3548560 (ChEMBL metabolism graph) and Pa = 0.879 for DBMET00991 (DrugBank metabolism graph).

Thus, taking into account the metabolism of Phenytoin increases the chances for the identification of potentially harmful actions of the drug by the in silico prediction of toxic and adverse effects at the early stages of research.

#### 2.3.2. Mechanisms of Action Predicted for Clozapine and Its Metabolites

Clozapine is a well-known multitargeted drug that is launched as an antipsychotic for the treatment of schizophrenia [22]. According to the PASS 2019 training set, 67 different mechanisms of action are known for this pharmaceutical (Table 2).

Prediction for the parent structure of Clozapine at Pa > Pi threshold gives 54 of 67 known mechanisms of action with average Pa = 0.19. If the structures of three Clozapine’s metabolites taken from the DrugBank metabolism graph are taken into consideration, at the same threshold, 59 of 67 known mechanisms of action are predicted with an average Pa_max = 0.21. Five activities additionally predicted for the metabolites’ structures include adenosine A3 receptor antagonist, adenylate cyclase inhibitor, adrenaline agonist, immunomodulator, and transcription factor NF kappa B inhibitor. They are marked in bold in Table 1.

Since the number of metabolites described in the ChEMBL database is relatively small, we performed an additional informational search in the Metabolite database [23]. We carried out predictions for 95 Clozapine metabolites found in this database and compared the results with the experimentally determined mechanisms of action. In this case, 62 of 67 mechanisms of action were predicted with an average Pa_max = 0.26. Three additional predicted activities (acetylcholine M3 receptor antagonist, adrenaline uptake inhibitor, and peptidyl-prolyl cis-trans isomerase inhibitor) are marked in bold and italic in Table 2.

Thus, taking into account information on Clozapine’s metabolites, increases the accuracy of the computer-aided estimates of the drug’s mechanisms of action. The quality of prediction increases if more data about the metabolites is available.

## 3. Discussion

Since most pharmaceuticals are metabolized in the human body, the observed biological effects are caused by integral actions of both the parent substance and its metabolites [4,24]. In the case of prodrugs, metabolites produce the desirable pharmacotherapeutic effect after biotransformation, and ordinarily, prodrugs are created to improve the pharmacokinetics of active pharmaceutical ingredients. Besides, metabolites may exhibit pharmacological effects that are similar to the parent substance, but less potent. Earlier, we demonstrated that in 74% of cases, metabolites’ biological activity may be predicted based on the structure of the parent compounds [25].

Unfortunately, some metabolites also cause adverse or toxic effects, restricting the application of particular pharmaceuticals in medicine. To reduce the risks of failed projects at the late stage of drug development or even rejection of the launched medicine from the market, it is necessary to identify the undesirable effects of the investigational drug as early as possible.

Many in silico methods are currently available to predict the pharmaceutical agents’ interactions with molecular targets and biological effects induced by such interactions (see, e.g., [7,8,9,10,26,27,28,29]). Such methods estimate the biological activity profiles with reasonable accuracy based on the structural formula of drug-like compounds. Similarly, computational methods are used to predict drug metabolism (see, e.g., [4,11,12,13,30,31,32]). These methods predict (a) the interaction of the drug substance with particular biotransformation enzymes, (b) the most probable sites of biotransformation, (c) the most probable metabolites.

In this study, we estimated if the accuracy of predicted biological activity profiles could be increased by considering the structures of both the parent substance and its metabolites. Information about the structural formulae of drugs’ metabolites was extracted from the publicly available biotransformation schemes presented in DrugBank [16] and ChEMBL [17]. Prediction of biological activity profiles has been performed by the computer program PASS (see Section 4.1), which provides the assessment of known biological activities with reasonable accuracy—exceeding those of many other available (Q)SAR methods [33].

It was demonstrated that the average values of both quality metrics, precision and recall, are increased for each of the four categories of activity (pharmacological effects, mechanisms of action, toxic and adverse effects, and antitargets). The most significant increase for the recall values is detected if the smallest Pa threshold is selected, while for the precision values, the opposite tendency is observed. The best-balanced accuracy is achieved for Pa values in the range 0.4–0.5. For the toxic and adverse effects at Pa = 0.5 threshold the maximum increase in quality of prediction was found; thus, adding the information about metabolites’ predicted activity profile leads to filtering out the potentially harmful drug-candidates. This general conclusion is illustrated by predicting the toxic and adverse effects for the anticonvulsant drug Phenytoin, for which a relatively narrow therapeutic index is experimentally determined.

For the well-known multitargeted drug Clozapine, we found that the more complete the data on its metabolites, the higher the prediction quality reached. This example demonstrates the main limitation of the proposed approach, associated with incomplete information about metabolites in the available databases. As we found in some cases, metabolic schemes are different in ChEMBL, DrugBank, and Metabolite databases, scrupulous collection of information and the prior manual curation of the metabolism data is necessary to obtain a more reliable computational assessment of the integral biological activity profiles. Utilization of the in silico predicted metabolic reactions and generated metabolites’ structures could also enhance the assessment, if filtering out potential “false positives” is provided [34].

Based on the results obtained in this study, we have developed a web server MetaPASS that allows for the prediction of the biological activity profiles for the parent substances of a drug and its metabolites, and integrates the obtained results for the complex assessment of the biological action and safety [35]. In addition to the biotransformation schemes extracted from DrugBank and ChEMBL, the user may upload the in house prepared scheme and then obtain the predictions. 

## 4. Materials and Methods

### 4.1. Prediction of Activity Spectra for Substances (PASS)

Currently, computational methods are widely used in drug discovery and development [36,37,38]. The increasing number of available chemical compounds [39,40,41] and the number of known molecular targets [42,43] and different pharmacotherapeutic effects [44], lead to the enormous space of chemical–biological interactions. Experimental testing of the interactions of many millions of chemical compounds with thousands of molecular targets is impossible from both an economical and practical point of view [45]. Thus, there is a need for prior selection of the molecules most probably interacting with the particular target(s) and exhibiting, due to this interaction, the necessary pharmacotherapeutic effect(s).

PASS development started in the framework of the State Registration System of New Chemical Compounds Synthesized in the USSR [46]. At that time, most of the pharmacological investigations were performed in vivo using experimental animals. Thus, the ligand-based approach implemented in the PASS, was initially based on the training set, which included data about biological activity obtained in complex biological systems. Therefore, it was necessary to develop a method that allowed us to analyze the structure–activity relationships (SAR) for pharmacological effects that arise due to the interaction of the parent pharmaceutical substance and its metabolites with the whole organism.

We proposed the concept of the Biological Activity Spectrum (Profile), which represents the set of different biological activities, resulting from the interaction of drug-like compounds with various biological objects. The Biological Activity Spectrum (BAS) reflects the “internal” properties of the compound, which depend only on its structure. BAS includes pharmacological effects, molecular mechanisms of action, specific toxicity and side effects, metabolism, and effects on undesirable targets, molecular transport, and gene expression. For each particular biological activity, every compound in the PASS training set is classified as “active” or “inactive,” depending on the available information. When the quantitative data are available, the compound is considered "active" if the semi-effective concentration is less than 10 μM. If information about the biological activity is not found for a compound, it is assumed that the compound is “inactive”. This approximation does not affect the results of the analysis of the structure–activity relationship significantly due to the robustness of the SAR method used in PASS [47]. It provides an opportunity to combine in the PASS training set, large amounts of data about biological activity extracted from many sources.

The description of the molecular structure of drug-like organic compounds is based on its structural formula; since this is the only available information at the early stages of research, (the compound may be only designed on a computer and planned for synthesis). To analyze SAR with PASS, in 1999 we proposed the Multilevel Neighborhoods of Atoms (MNA) descriptors, which reflects the important features of local correspondence in ligand–target interactions [48]. MNA descriptors are remarkably close to the very popular Extended-Connectivity Fingerprints (ECFP) that were published in 2006 [49].

Like any other ligand-based drug design approach, PASS requires the training sets to analyze the SAR. In the PASS 2019 version, the training set includes information on 1,025,468 structural formulae of drug-like molecules and about 8054 types of biological activity (totally, over 8 billion entries). In this study, we applied the PASS Refined version that is limited to 1333, the most important biological activities predicted with the highest accuracy. For some particular purposes, we have created the specialized versions of PASS based on different training sets. For instance, the web service ADVERPred that predicts such adverse effects of drug-like compounds as myocardial infarction, arrhythmia, cardiac failure, hepatotoxicity, and nephrotoxicity is based on SAR identified by PASS from the training set compiled from the relevant sources [21].

During the training procedure for the elucidation of SAR and the prediction of biological activity spectra of new compounds by PASS, the modified version of the Bayesian approach is applied. 

As a result of PASS prediction, one obtains the list of probable activities, Pa that is the probability of belonging to the class of “actives”, and Pi that is the probability of belonging to the class of “inactives”. For choosing a threshold in the classification problem, the probabilities Pa and Pi are also, by construction, the probabilities of the first and second kinds of prediction error, respectively, and 1-Pa and 1-Pi are estimates of sensitivity and specificity. By default, the threshold is Pa = Pi (or Sensitivity = Specificity), so all activities with Pa > Pi are considered as probable.

For each of the activities, the prediction accuracy is estimated in PASS as the probability that for an arbitrary pair of active and inactive compounds, the Pa value for the active compound will be higher than the Pa value for the inactive compound. It is called Invariant Accuracy of Prediction (IAP) [7,14] and equals to the area under the receiver operating characteristic curve (AUC ROC) criterion. In the leave-one-out cross-validation procedure for the PASS Refined version average IAP = 0.9717. Thus, PASS provides both a high accuracy of SAR determined based on the training set and reasonable predictivity for a new compound. 

For a more detailed description of the PASS approach, see [7,14,15].

In this study, all the information about the 864 drug substances analyzed was excluded from the PASS training set before the prediction, carried out for both the parent molecule and its metabolites.

### 4.2. Sources of Information for Creation of the Metabolic Graphs

In this work, we have used the two most known, free-to-access online databases containing information on biotransformation reactions and structures of substrates and biotransformation products.

**ChEMBL** [17,50] is a comprehensive database containing information on bioactive molecules with drug-like properties, and which is created and maintained by the EMBL—EBI (European Molecular Biology Laboratory—European Bioinformatics Institute, UK). ChEMBL may be downloaded in different formats, including dump files for database management systems, such as Oracle, MySQL, and PostgreSQL. The ChEMBL database (version 26) holds information about two million compounds, including 1245 records of biotransformation (for 168 parent compounds), including the enzyme name, explanation of conversion, and the structures of metabolites. Information on the substrate–metabolite pairs was taken from the “METABOLISM” table. The chemical structures were extracted from the “COMPOUND_STRUCTURES” table.

**DrugBank** [16,51] is a comprehensive database containing information on drugs and drug targets. The DrugBank database may be downloaded in XML and SDF formats. The DrugBank 5.1 version holds data for more than 2500 drugs, including 3300 records of biotransformation (for 872 parent compound), including the enzyme name, type of reactions, and the structures of metabolites. Information on the substrate-metabolite pairs was taken from an XML file, where the “<reaction>” tag consists of “<left-element>” and “<right-element>”, which correspond to the substrate and metabolite IDs. The chemical structures were extracted from *.sdf files.

We have combined information on biotransformation from these databases. After the compounds’ structures were normalized (salts removed) and the same compounds with the same metabolic pathways were removed, 142 parent compounds from ChEMBL and 822 parent compounds from DrugBank remained in MetaPASS.

### 4.3. Integral Biological Activity Spectra Assessment

For an integral assessment of the spectrum of biological activity in MetaPASS, we used an assessment of the most probable activity estimate for the parent compound and its metabolites. Such an approach corresponds to the concept of the biological activity spectrum [7,14], which suggests that a compound exhibits a particular type of biological activity if there are some experimental conditions at which this activity is observed.

### 4.4. (Q)SAR Quality Metrics 

Two parameters “Precision” and “Recall” were used as the quality metrics of predictions. They were calculated according to the formulae:

$$Precision=\frac{TP}{TP+FP},\;\;Recall=\frac{TP}{TP+FN},$$

where: *TP* is the true positives corresponding to the number of predicted known activities; *FP* is the false positives corresponding to the number of predicted but unknown activities; *FN* is the false negatives corresponding to the known but unpredicted activities.

## 5. Conclusions

Modern chemoinformatics methods are widely used to predict biological activity profiles and metabolic transformations of novel pharmaceutical agents under study as potential drug candidates. It is known that the biological activity of drug metabolites may differ from those of the parent compounds, which sometimes leads to the rejection of the launched medicines from the market. Thus, the complex assessment of biological activity profiles, demonstrated by structures of both the parent compound and its metabolites at the early stage of drug research, may help identify the essential issues that influence the project’s success or failure.

In this study, we investigated if a combination of the in silico prediction of biological activity spectra with the computer program PASS and the existing knowledge about the metabolism of drugs may increase predictions’ accuracy. Metabolism graphs have been developed based on the information about biotransformation schemes available from ChEMBL and DrugBank. It was shown that the integration of data on biological activity profiles estimated for the parent compounds and its metabolites increase both precision and recall parameters, which characterized the accuracy of prediction. The best-balanced estimates are obtained when Pa about 0.5 values are used as the threshold. Such results are observed for each of the four categories of activity, including pharmacological effects, mechanisms of action, toxic and adverse effects, and antitargets. Since the most substantial improvement is observed for the toxic and adverse effects, the obtained results may help filter out the cases when potentially hazardous metabolites could be expected. 

In case studies for Phenytoin and Clozapine, we demonstrated that taking into account drug metabolism might enrich the predicted biological activity profiles. Such an integral assessment could be used not only to identify toxic and adverse effects but also for extension of the possible therapeutic applications of the investigational drugs.

A web service MetaPASS, developed based on the results obtained in this study, will allow for determining the possible directions for repurposing the launched drugs, optimizing the selectivity, and estimating new pharmaceutical agents’ safety.

## Figures and Tables

**Table 1:** Toxic and adverse effects predicted for Phenytoin (Pa) and the highest Pa_max estimates for its metabolites.

Pa	Pa_Max	Activity
**0.534**	**0.844**	**Teratogen**
**0.571**	**0.831**	**Carcinogenic**
**0.495**	**0.829**	**Embryotoxic**
**0.39**	**0.795**	**Sedative**
**0.277**	**0.785**	**Carcinogenic, mouse, female**
**0.278**	**0.781**	**Carcinogenic, mouse**
**0.13**	**0.701**	**Mutagenic**
**0.531**	**0.694**	**Carcinogenic, group 2B**
0.224	0.549	Ulceration
0.185	0.506	Lacrimal secretion stimulant
0.284	0.483	Carcinogenic, group 1
0.255	0.356	Pneumotoxic
0.23	0.325	Carcinogenic, rat, male
0.192	0.322	Carcinogenic, group 2A
0.213	0.313	Carcinogenic, rat

**Table 2:** Clozapine’s mechanisms of action according to the PASS training set.

No	Activity *	No	Activity *
1	5 Hydroxytryptamine 1 antagonist	35	Alpha 2 adrenoreceptor antagonist
2	5 Hydroxytryptamine 1A antagonist	36	Alpha 2a adrenoreceptor antagonist
3	5 Hydroxytryptamine 1B antagonist	37	Alpha 2b adrenoreceptor antagonist
4	5 Hydroxytryptamine 1D antagonist	38	Alpha 2c adrenoreceptor antagonist
5	5 Hydroxytryptamine 2 antagonist	39	Alpha adrenoreceptor agonist
6	5 Hydroxytryptamine 2A antagonist	40	Alpha adrenoreceptor antagonist
7	5 Hydroxytryptamine 2B antagonist	41	Analgesic
8	5 Hydroxytryptamine 2C antagonist	42	Antiadrenergic
9	5 Hydroxytryptamine 3 antagonist	42	Antihistaminic
10	5 Hydroxytryptamine 6 antagonist	44	Cholinergic antagonist
11	5 Hydroxytryptamine 7 antagonist	45	Dopamine D1 antagonist
12	5 Hydroxytryptamine agonist	46	Dopamine D2 antagonist
13	5 Hydroxytryptamine antagonist	47	Dopamine D3 antagonist
14	Acetylcholine agonist	48	Dopamine D4 agonist
15	Acetylcholine antagonist	49	Dopamine D4 antagonist
16	Acetylcholine M1 receptor agonist	50	GABA A receptor antagonist
17	Acetylcholine M1 receptor antagonist	51	GABA receptor antagonist
18	Acetylcholine M2 receptor antagonist	52	Histamine agonist
19	***Acetylcholine M3 receptor antagonist***	53	Histamine antagonist
20	Acetylcholine M4 receptor antagonist	54	Histamine H1 receptor antagonist
21	Acetylcholine M5 receptor antagonist	55	Histamine H2 receptor antagonist
22	Acetylcholine muscarinic agonist	56	Histamine H3 receptor antagonist
23	Acetylcholine muscarinic antagonist	57	Histamine H4 receptor agonist
24	**Adenosine A3 receptor antagonist**	58	Histamine H4 receptor antagonist
25	**Adenylate cyclase inhibitor**	59	**Immunomodulator**
26	**Adrenaline agonist**	60	Opioid agonist
27	Adrenaline antagonist	61	Opioid delta receptor agonist
28	***Adrenaline uptake inhibitor***	62	***Peptidyl-prolyl cis-trans isomerase inhibitor***
29	Alpha 1 adrenoreceptor agonist	63	Potassium channel (Voltage-sensitive) blocker
30	Alpha 1 adrenoreceptor antagonist	64	Potassium channel blocker
31	Alpha 1a adrenoreceptor antagonist	65	Sigma receptor antagonist
32	Alpha 1b adrenoreceptor antagonist	66	Transcription factor inhibitor
33	Alpha 1d adrenoreceptor antagonist	67	**Transcription factor NF kappa B inhibitor**
34	Alpha 2 adrenoreceptor agonist		

* Some results look contradictory because, at different drug concentrations, the agonistic or antagonistic action on the same receptor may be exhibited.

## Supplementary Materials

Supplementary materials can be found at https://www.mdpi.com/1422-0067/21/20/7492/s1.
