# Supplementary material for: Computer-Aided Estimation of Biological Activity Profiles of Drug-Like Compounds Taking into Account Their Metabolism in Human Body
Source: Int J Mol Sci. 2020 Oct 11;21(20):7492. doi: 10.3390/ijms21207492 (PMC7593915; doi:10.3390/ijms21207492)
Supplement: Supplementary file 1 [file ijms-21-07492-s001.zip › Filimonov_DA-et-al-Table_S5.docx]

**Table S5.** Lists of biological activities belonging to the category “Antitargets”. NA is the number of active compounds; IAP is an Invariant Accuracy of Prediction obtained in leave-one-out cross-validation.

| **Activity** | **NA** | **IAP, LOO CV** |
| --- | --- | --- |
| HERG channel blocker | 4273 | 0.9420 |
| GRP78 expression inhibitor | 5 | 0.9999 |
| CYP1A2 inhibitor | 807 | 0.9022 |
| CYP2C9 inhibitor | 3053 | 0.8789 |
| CYP2D6 inhibitor | 3513 | 0.9101 |
| CYP3A4 inhibitor | 6463 | 0.8971 |
| 11-Beta-hydroxysteroid dehydrogenase 2 inhibitor | 299 | 0.9966 |
| 5 Hydroxytryptamine 1A agonist | 1246 | 0.9923 |
| 5 Hydroxytryptamine 1B agonist | 114 | 0.9941 |
| 5 Hydroxytryptamine 2A agonist | 196 | 0.9850 |
| 5 Hydroxytryptamine 2B agonist | 112 | 0.9848 |
| 5 Hydroxytryptamine 2C antagonist | 1938 | 0.9874 |
| 5 Hydroxytryptamine 3 agonist | 114 | 0.9844 |
| 5 Hydroxytryptamine 4 agonist | 525 | 0.9947 |
| ATPase inhibitor | 155 | 0.9394 |
| Acetylcholine M1 receptor agonist | 1647 | 0.9928 |
| Acetylcholine M1 receptor antagonist | 2149 | 0.9524 |
| Acetylcholine M2 receptor agonist | 204 | 0.9860 |
| Acetylcholine M2 receptor antagonist | 2064 | 0.9902 |
| Acetylcholine M3 receptor agonist | 121 | 0.9838 |
| Acetylcholine M3 receptor antagonist | 2197 | 0.9904 |
| Acetylcholinesterase inhibitor | 3713 | 0.9719 |
| Aconitate hydratase inhibitor | 22 | 0.9933 |
| Acyl-CoA dehydrogenase inhibitor | 15 | 0.9953 |
| Adenosine A1 receptor agonist | 539 | 0.9976 |
| Adenosine A2a receptor antagonist | 2851 | 0.9870 |
| Adenosine deaminase inhibitor | 249 | 0.9909 |
| Adenylate cyclase I inhibitor | 54 | 0.9980 |
| Adenylate cyclase inhibitor | 207 | 0.9503 |
| Adenylate kinase inhibitor | 26 | 0.9456 |
| Alcohol dehydrogenase inhibitor | 151 | 0.9664 |
| Aldosterone antagonist | 226 | 0.9839 |
| Alkaline phosphatase inhibitor | 419 | 0.9496 |
| Alpha 1a adrenoreceptor agonist | 140 | 0.9995 |
| Alpha 1a adrenoreceptor antagonist | 3345 | 0.9859 |
| Alpha 1b adrenoreceptor antagonist | 1449 | 0.9873 |
| Alpha 2a adrenoreceptor agonist | 24 | 0.9970 |
| Alpha 2a adrenoreceptor antagonist | 724 | 0.9827 |
| Alpha 2b adrenoreceptor agonist | 86 | 0.9905 |
| Alpha-mannosidase inhibitor | 64 | 0.9881 |
| Aminopeptidase A inhibitor | 67 | 0.9995 |
| Aminopeptidase N inhibitor | 481 | 0.9890 |
| Aminopeptidase P inhibitor | 10 | 1.0000 |
| Androgen agonist | 633 | 0.9830 |
| Androgen antagonist | 1855 | 0.9790 |
| Arginase inhibitor | 81 | 0.9973 |
| Argininosuccinate synthase inhibitor | 21 | 0.9927 |
| Aryl hydrocarbon receptor agonist | 125 | 0.9114 |
| Beta 1 adrenoreceptor agonist | 778 | 0.9966 |
| Beta 1 adrenoreceptor antagonist | 872 | 0.9896 |
| Beta 2 adrenoreceptor agonist | 2308 | 0.9745 |
| Beta 2 adrenoreceptor antagonist | 884 | 0.9910 |
| Bradykinin B1 receptor agonist | 8 | 1.0000 |
| Bradykinin B2 receptor agonist | 9 | 0.9999 |
| Butyrylcholinesterase inhibitor | 1734 | 0.9863 |
| Ca2+-transporting ATPase inhibitor | 12 | 0.8723 |
| Calcium channel L-type blocker | 2108 | 0.9873 |
| Cannabinoid CB1 receptor antagonist | 2616 | 0.9831 |
| Cannabinoid CB2 receptor antagonist | 1223 | 0.9916 |
| Carbamoyl-phosphate synthase (ammonia) inhibitor | 15 | 0.9942 |
| Carbonic anhydrase I inhibitor | 2450 | 0.9929 |
| Carbonic anhydrase II inhibitor | 3148 | 0.9928 |
| Carbonic anhydrase inhibitor | 3698 | 0.9919 |
| Catalase inhibitor | 69 | 0.9886 |
| Catechol O methyltransferase inhibitor | 199 | 0.9923 |
| Cyclooxygenase 1 inhibitor | 2126 | 0.9731 |
| Cyclooxygenase 2 inhibitor | 4879 | 0.9751 |
| Cyclooxygenase inhibitor | 6529 | 0.9660 |
| Cystathionine beta-synthase inhibitor | 12 | 0.9920 |
| DOPA decarboxylase inhibitor | 12 | 0.9540 |
| Diamine oxidase inhibitor | 135 | 0.9946 |
| Dihydrofolate reductase inhibitor | 2608 | 0.9926 |
| Dipeptidyl peptidase IV inhibitor | 3423 | 0.9938 |
| Dopamine D2 agonist | 528 | 0.9936 |
| Dopamine D2 antagonist | 5530 | 0.9849 |
| Endothelin A receptor antagonist | 2172 | 0.9980 |
| Estrogen agonist | 1383 | 0.9885 |
| Fumarate hydratase inhibitor | 24 | 0.9974 |
| GABA A receptor agonist | 745 | 0.9771 |
| GABA A receptor antagonist | 3146 | 0.9893 |
| GABA aminotransferase inhibitor | 43 | 0.9678 |
| Glucocorticoid agonist | 901 | 0.9972 |
| Glucocorticoid antagonist | 1512 | 0.9955 |
| Glucose-6-phosphate isomerase inhibitor | 23 | 0.9992 |
| Glutamate dehydrogenase inhibitor | 36 | 0.9699 |
| HMG CoA reductase inhibitor | 1366 | 0.9969 |
| Heat shock protein 70 antagonist | 23 | 0.9650 |
| Hexokinase inhibitor | 271 | 0.9520 |
| Histamine H1 receptor agonist | 53 | 0.9857 |
| Histamine H1 receptor antagonist | 1710 | 0.9842 |
| Histamine H2 receptor agonist | 60 | 0.9742 |
| Histamine H2 receptor antagonist | 604 | 0.9846 |
| Histamine H3 receptor agonist | 313 | 0.9963 |
| Hypoxanthine phosphoribosyltransferase inhibitor | 79 | 0.9964 |
| Lck kinase inhibitor | 3408 | 0.9642 |
| Lysine carboxypeptidase inhibitor | 35 | 0.9983 |
| MAO A inhibitor | 1454 | 0.9821 |
| MAO inhibitor | 2771 | 0.9741 |
| NMDA receptor agonist | 181 | 0.9843 |
| Na+ K+ transporting ATPase inhibitor | 187 | 0.9890 |
| Nav1.5 sodium channel blocker | 278 | 0.9644 |
| Neuropeptide Y1 antagonist | 503 | 0.9392 |
| Neutral endopeptidase inhibitor | 1401 | 0.9972 |
| Nicotinic alpha4 receptor agonist | 93 | 0.9997 |
| Opioid delta receptor agonist | 1302 | 0.9935 |
| Opioid delta receptor antagonist | 2699 | 0.9893 |
| Opioid kappa receptor agonist | 1178 | 0.9906 |
| Opioid mu receptor agonist | 1133 | 0.9909 |
| Ornithine carbamoyltransferase inhibitor | 40 | 0.9935 |
| Peroxidase inhibitor | 16 | 0.9433 |
| Phenylalanine 4-hydroxylase inhibitor | 34 | 0.9911 |
| Phosphodiesterase inhibitor | 10558 | 0.9682 |
| Phosphofructokinase-1 inhibitor | 33 | 0.9902 |
| Phosphoglycerate kinase inhibitor | 189 | 0.9969 |
| Phospholipase A2 inhibitor | 1606 | 0.9467 |
| Phospholipase C inhibitor | 81 | 0.9602 |
| Phosphorylase inhibitor | 602 | 0.9913 |
| Platelet activating factor antagonist | 6895 | 0.9716 |
| Prostaglandin F2 alpha agonist | 89 | 0.9879 |
| Pyruvate kinase inhibitor | 492 | 0.9374 |
| Retinoic acid alpha receptor agonist | 75 | 0.9993 |
| S-adenosyl-L-homocysteine hydrolase inhibitor | 167 | 0.9997 |
| Sodium channel blocker | 5445 | 0.9657 |
| Succinate dehydrogenase inhibitor | 14 | 0.9606 |
| Superoxide dismutase inhibitor | 16 | 0.9067 |
| Thyroid hormone agonist | 165 | 0.9924 |
| Thyroid hormone alpha agonist | 39 | 0.9999 |
| Thyroid hormone beta agonist | 111 | 0.9998 |
| Topoisomerase I inhibitor | 874 | 0.9775 |
| Triose-phosphate isomerase inhibitor | 23 | 0.9909 |
| Tyrosine 3 hydroxylase inhibitor | 12 | 0.8985 |
| UDP-glucose 4-epimerase inhibitor | 28 | 0.9762 |
| Excitatory amino acid transporter 2 inhibitor | 56 | 0.9997 |
| GABA transporter 1 inhibitor | 245 | 0.9945 |
| 5 Hydroxytryptamine uptake inhibitor | 5503 | 0.9880 |
| Adrenaline uptake inhibitor | 3215 | 0.9901 |
| Dopamine uptake inhibitor | 1907 | 0.9941 |
| Electron transport complex I inhibitor | 78 | 0.9943 |
