# Supplementary material for: Computer-Aided Estimation of Biological Activity Profiles of Drug-Like Compounds Taking into Account Their Metabolism in Human Body
Source: Int J Mol Sci. 2020 Oct 11;21(20):7492. doi: 10.3390/ijms21207492 (PMC7593915; doi:10.3390/ijms21207492)
Supplement: Supplementary file 1 [file ijms-21-07492-s001.zip › Filimonov_DA-et-al-Table_S6.docx]

**Table S6.** The list of activities predicted for Phenytoin and its metabolites*.

| **Pa** | **Pi** | **Pa_max** | **Pi(ID Pa_max)** | **Activity** | **ID Pa_max** |
| --- | --- | --- | --- | --- | --- |
| **0.782** | **0.066** | **0.876** | **0.037** | **Hepatotoxicity** | **CHEMBL3548560** |
| 0.489 | 0.116 | 0.55 | 0.071 | Arrhythmia | CHEMBL3544501 |
| 0.121 | 0.534 | 0.402 | 0.084 | Nephrotoxicity | CHEMBL3544522 |
| 0.120 | 0.442 | 0.413 | 0.091 | Cardiac failure | CHEMBL3544524 |
| 0.121 | 0.534 | 0.297 | 0.179 | Myocardial infarction | CHEMBL3544524 |

* Prediction is carried out with ADVERPred software [1].

1. Ivanov. S.M.; Lagunin, A.A.; Rudik, A.V., Filimonov, D.A.; Poroikov, V.V. ADVERPred – web service for prediction of adverse effects of drugs. *J. Chem. Inf. Model*., **2018**, *58*, 8-11. [[CrossRef](http://dx.doi.org/10.1021/acs.jcim.7b00568)] [[PubMed](https://pubmed.ncbi.nlm.nih.gov/29206457/)]
