# Supplementary material for: Computer-Aided Estimation of Biological Activity Profiles of Drug-Like Compounds Taking into Account Their Metabolism in Human Body
Source: Int J Mol Sci. 2020 Oct 11;21(20):7492. doi: 10.3390/ijms21207492 (PMC7593915; doi:10.3390/ijms21207492)
Supplement: Supplementary file 1 [file ijms-21-07492-s001.zip › Filimonov_DA-et-al-Table_S1.docx]

**Table S1.** The list of the analyzed pharmaceutical substances. 782 drug substances, which (1) contain the structural formulas of metabolites and (2) are included in PASS training set, are highlighted in bold. The number of known metabolites is shown in parentheses after the compound’s identifier.

| **No.** | **Compound’s name** | **Identifier in ChEMBL or DrugBank** |
| --- | --- | --- |
| **1** | **(2R)-1-(2,6-dimethylphenoxy)propan-2-amine** | **DB07129(4)** |
| **2** | **(4R)-limonene** | **DB08921(5)** |
| **3** | **(R)-fluoxetine** | **DB08472(3)** |
| **4** | **(S)-camphor** | **DB11345(1)** |
| **5** | **(S)-fluoxetine** | **DB08544(3)** |
| **6** | **Abiraterone** | **DB05812(2)** |
| 7 | Acalabrutinib | DB11703(13) |
| **8** | **Aceclofenac** | **DB06736(5)** |
| **9** | **Acemetacin** | **DB13783(7)** |
| **10** | **Acenocoumarol** | **DB01418(3)** |
| **11** | **Acetaminophen** | **CHEMBL112 (5); DB00316 (4)** |
| **12** | **Acetohexamide** | **DB00414(1)** |
| **13** | **Acetylsalicylic acid** | **DB00945(5)** |
| 14 | Acyclovir | CHEMBL184 (2); DB00787(2) |
| **15** | **Adefovir dipivoxil** | **DB00718(3)** |
| **16** | **Ademetionine** | **DB00118(5)** |
| **17** | **Adenosine** | **DB00640(2)** |
| **18** | **Adinazolam** | **DB00546(3)** |
| **19** | **Albendazole** | **DB00518(2)** |
| **20** | **Aldosterone** | **DB04630(2)** |
| **21** | **Alectinib** | **DB11363(4)** |
| **22** | **Alfentanil** | **DB00802(4)** |
| **23** | **Aliskiren** | **CHEMBL1639(10)** |
| **24** | **Allopurinol** | **CHEMBL859(7); DB00437(1)** |
| **25** | **Almotriptan** | **DB00918(5)** |
| **26** | **Alpelisib** | **DB12015(1)** |
| **27** | **Alpha-Tocopherol succinate** | **DB14001(1)** |
| **28** | **Alprazolam** | **CHEMBL661(7); DB00404(5)** |
| **29** | **Alprenolol** | **DB00866(1)** |
| **30** | **Altretamine** | **CHEMBL1455(8)** |
| **31** | **Alvespimycin** | **CHEMBL383824(6)** |
| **32** | **Ambrisentan** | **CHEMBL383824(6); CHEMBL1111(8)** |
| **33** | **Amifampridine** | **DB11640(1)** |
| **34** | **Aminocaproic acid** | **CHEMBL1046(1); DB00513(1)** |
| **35** | **Aminophenazone** | **DB01424(2)** |
| **36** | **Amiodarone** | **CHEMBL633(1); DB01118(2)** |
| **37** | **Amitriptyline** | **CHEMBL445(12); DB00321(5)** |
| 38 | Amlodipine | DB00381(2) |
| **39** | **Amodiaquine** | **DB00613(1)** |
| **40** | **Amoxapine** | **DB00543(2)** |
| **41** | **Amoxicillin** | **DB01060(7)** |
| **42** | **Amphetamine** | **DB00182(6)** |
| **43** | **Ampicillin/ampicillin trihydrate** | **CHEMBL174(1)** |
| 44 | Amprenavir | CHEMBL116(5) |
| **45** | **Amrubicin** | **DB06263(3)** |
| **46** | **Anagrelide** | **DB00261(2)** |
| **47** | **Anastrozole** | **CHEMBL445(12); DB00321(5)** |
| **48** | **Androstenedione** | **DB01536(1)** |
| **49** | **Anethole trithione** | **DB13853(1)** |
| 50 | Angiotensin II | DB11842(2) |
| **51** | **Aniline** | **DB06728(2)** |
| **52** | **Antipyrine** | **DB01435(4)** |
| **53** | **Apalutamide** | **DB11901(1)** |
| **54** | **Apixaban** | **DB06605(1)** |
| **55** | **Apremilast** | **DB05676(2)** |
| **56** | **Aprepitant** | **DB00673(7)** |
| **57** | **Aprindine** | **DB01429(3)** |
| **58** | **Arachidonic acid** | **DB04557(18)** |
| **59** | **Aranidipine** | **DB09229(5)** |
| 60 | Arbutamine | DB01102(2) |
| **61** | **Arbutin** | **DB11217(4)** |
| **62** | **Aripiprazole** | **DB01238(16)** |
| **63** | **Aripiprazole lauroxil** | **DB14185(4)** |
| 64 | Arsenic trioxide | DB01169(4) |
| **65** | **Artemether** | **DB06697(5)** |
| **66** | **Artenimol** | **DB11638(1)** |
| **67** | **Arverapamil** | **DB06669(3)** |
| **68** | **Ascorbic acid** | **DB00126(6)** |
| **69** | **Aspirin** | **CHEMBL25(8)** |
| **70** | **Astemizole** | **DB00637(4)** |
| **71** | **Asunaprevir** | **DB11586(5)** |
| **72** | **Ataluren** | **DB05016(1)** |
| **73** | **Atazanavir** | **CHEMBL1163(23)** |
| **74** | **Atenolol** | **CHEMBL24(1); DB00335(2)** |
| **75** | **Atomoxetine** | **DB00289(4)** |
| **76** | **Atorvastatin** | **DB01076(7)** |
| **77** | **Atosiban** | **DB09059(3)** |
| **78** | **Azathioprine** | **CHEMBL1542(9); DB00993(11)** |
| **79** | **Azelastine** | **DB00972(1)** |
| 80 | Baclofen | DB00181(1) |
| **81** | **Balsalazide** | **DB01014(2)** |
| **82** | **Bambuterol** | **DB01408(1)** |
| **83** | **Barnidipine** | **DB09227(5)** |
| 84 | Bazedoxifene | DB06401(2) |
| **85** | **Beclomethasone dipropionate** | **DB00394(3)** |
| **86** | **Belinostat** | **DB05015(1)** |
| 87 | Bempedoic acid | DB11936(2) |
| **88** | **Benazepril** | **DB00542(1)** |
| **89** | **Bendamustine** | **DB06769(4)** |
| **90** | **Bendazac** | **DB13501(1)** |
| **91** | **Benidipine** | **DB09231(2)** |
| **92** | **Benserazide** | **DB12783(1)** |
| **93** | **Benzatropine** | **DB00245(5)** |
| 94 | Benzhydrocodone | DB15465(1) |
| **95** | **Benzoic acid** | **DB03793(1)** |
| **96** | **Benzonatate** | **DB00868(1)** |
| **97** | **Benzoyl peroxide** | **DB09096(2)** |
| **98** | **Benzphetamine** | **DB00865(2)** |
| **99** | **Benzydamine** | **DB09084(2)** |
| **100** | **Benzylpenicillin** | **CHEMBL29(7); DB01053(2)** |
| **101** | **Beta carotene** | **DB06755(2)** |
| **102** | **Betahistine** | **DB06698(1)** |
| **103** | **Betrixaban** | **DB12364(7)** |
| **104** | **Bimatoprost** | **DB00905(1)** |
| **105** | **Biotin** | **DB00121(2)** |
| **106** | **Bisacodyl** | **CHEMBL942(2)** |
| **107** | **Blonanserin** | **DB09223(8)** |
| **108** | **Bortezomib** | **DB00188(15)** |
| **109** | **Bosentan** | **DB00559(3)** |
| 110 | Brexpiprazole | DB09128(1) |
| 111 | Brigatinib | DB12267(1) |
| **112** | **Brimonidine** | **DB00484(2)** |
| **113** | **Brivanib** | **CHEMBL377300(14)** |
| **114** | **Brofaromine** | **DB13876(1)** |
| **115** | **Bronopol** | **DB13960(5)** |
| **116** | **Bropirimine** | **DB04168(2)** |
| **117** | **Budesonide** | **DB01222(6)** |
| **118** | **Bufuralol** | **DB06726(3)** |
| 119 | Bupivacaine | DB00297(1) |
| **120** | **Bupranolol** | **DB08808(1)** |
| **121** | **Buprenorphine** | **DB00921(4)** |
| **122** | **Bupropion** | **CHEMBL894(3); DB01156(4)** |
| **123** | **Buspirone** | **CHEMBL724(9); DB00490(5)** |
| **124** | **Busulfan** | **CHEMBL820(8)** |
| **125** | **Butalbital** | **DB00241(2)** |
| 126 | Butylscopolamine | DB09300(3) |
| 127 | Butyrfentanyl | DB09173(7) |
| **128** | **Cabazitaxel** | **DB06772(3)** |
| **129** | **Cabergoline** | **DB00248(1)** |
| **130** | **Caffeine** | **DB00201(8)** |
| **131** | **Calcitriol** | **CHEMBL846(2); DB00136(1)** |
| 132 | Calcium carbimide | DB09116(3) |
| **133** | **Camphor** | **DB01744(2)** |
| 134 | Canagliflozin | DB08907(2) |
| **135** | **Candesartan cilexetil** | **DB00796(4)** |
| **136** | **Capecitabine** | **DB01101(2)** |
| **137** | **Captopril** | **DB01197(1)** |
| **138** | **Carbamazepine** | **DB00564(9)** |
| **139** | **Carbidopa** | **DB00190(6)** |
| **140** | **Carisoprodol** | **CHEMBL1233(5); DB00395(1)** |
| **141** | **Carteolol** | **DB00521(1)** |
| **142** | **Carvedilol** | **DB01136(6)** |
| **143** | **Cbio** | **CHEMBL445990(1)** |
| **144** | **Cefapirin** | **DB01139(1)** |
| **145** | **Cefepime** | **DB01413(2)** |
| **146** | **Cefotaxime** | **DB00493(1)** |
| **147** | **Ceftaroline fosamil** | **DB06590(1)** |
| **148** | **Cefuroxime** | **DB01112(2)** |
| **149** | **Celecoxib** | **CHEMBL118(3); DB00482(3)** |
| 150 | Cerivastatin | DB00439(2) |
| **151** | **Cetyl alcohol** | **DB09494(1)** |
| **152** | **Cevimeline** | **DB00185(5)** |
| **153** | **Chloramphenicol succinate** | **DB07565(1)** |
| **154** | **Chloroquine** | **DB00608(3)** |
| **155** | **Chloroxylenol** | **DB11121(2)** |
| **156** | **Chlorpromazine** | **CHEMBL71(4); DB00477(6)** |
| **157** | **Chlorpropamide** | **CHEMBL498(4); DB00672(6)** |
| **158** | **Chlorquinaldol** | **DB13306(1)** |
| **159** | **Chlorzoxazone** | **DB00356(1)** |
| **160** | **Cholecalciferol** | **DB00169(3)** |
| **161** | **Cholic acid** | **DB02659(1)** |
| 162 | Choline C 11 | DB09277(1) |
| **163** | **Cilazapril** | **DB01340(1)** |
| 164 | Cimetidine | CHEMBL30(3) |
| **165** | **Cinacalcet** | **DB01012(2)** |
| **166** | **Cinnamyl alcohol** | **DB14186(1)** |
| **167** | **Cinnarizine** | **DB00568(6)** |
| **168** | **Ciprofloxacin** | **DB00537(4)** |
| **169** | **Cisapride** | **DB00604(4)** |
| 170 | Cisatracurium | DB00565(2) |
| **171** | **Citalopram** | **CHEMBL549(4); DB00215(5)** |
| **172** | **Cladribine** | **DB00242(1)** |
| **173** | **Clarithromycin** | **CHEMBL1741(4); DB01211(2)** |
| 174 | Clavulanic acid | DB00766(2) |
| **175** | **Clindamycin** | **DB01190(2)** |
| **176** | **Clobazam** | **DB00349(2)** |
| **177** | **Clofarabine** | **DB00631(1)** |
| **178** | **Clofazimine** | **DB00845(1)** |
| **179** | **Clomipramine** | **DB01242(13)** |
| **180** | **Clonazepam** | **DB01068(2)** |
| **181** | **Clonidine** | **DB00575(1)** |
| **182** | **Clopidogrel** | **DB00758(3)** |
| **183** | **Clorazepic acid** | **DB00628(3)** |
| **184** | **Clotrimazole** | **CHEMBL104(2)** |
| **185** | **Clozapine** | **DB00363(3)** |
| **186** | **Cocaine** | **DB00907(3)** |
| **187** | **Codeine** | **DB00318(5)** |
| 188 | Coenzyme M | DB09110(1) |
| **189** | **Colchicine** | **DB01394(2)** |
| **190** | **Coumarin** | **DB04665(3)** |
| **191** | **Cs-1036** | **CHEMBL3527223(2)** |
| **192** | **Curcumin** | **DB11672(1)** |
| 193 | Cyanocobalamin | DB00115(1) |
| **194** | **Cyclobenzaprine** | **DB00924(2)** |
| **195** | **Cyclophosphamide** | **CHEMBL88(15); DB00531(12)** |
| **196** | **Cyclosporine** | **CHEMBL160(13); DB00091(3)** |
| **197** | **Cyproterone acetate** | **DB04839(1)** |
| **198** | **Dabigatran etexilate** | **DB06695(5)** |
| **199** | **Dacomitinib** | **DB11963(1)** |
| **200** | **Dalfampridine** | **DB06637(2)** |
| **201** | **D-alpha-Tocopherol acetate** | **DB14002(1)** |
| **202** | **Danazol** | **CHEMBL1479(5); DB01406(2)** |
| **203** | **Dantrolene** | **DB01219(1)** |
| **204** | **Dapagliflozin** | **DB06292(3)** |
| **205** | **Dapsone** | **DB00250(5)** |
| 206 | Darolutamide | DB12941(1) |
| **207** | **Daunorubicin** | **CHEMBL178(1)** |
| **208** | **Debrisoquine** | **DB04840(1)** |
| **209** | **Deflazacort** | **DB11921(2)** |
| **210** | **Delamanid** | **DB11637(8)** |
| **211** | **Delavirdine** | **DB00705(1)** |
| **212** | **Deltamethrin** | **DB13600(1)** |
| **213** | **Desipramine** | **DB01151(2)** |
| **214** | **Desogestrel** | **DB00304(12)** |
| **215** | **Desvenlafaxine** | **DB06700(4)** |
| 216 | Dexamethasone | DB01234(1) |
| **217** | **Dexchlorpheniramine** | **CHEMBL1201353(2)** |
| **218** | **Dexibuprofen** | **DB09213(2)** |
| **219** | **Dexlansoprazole** | **DB05351(1)** |
| **220** | **Dexmethylphenidate** | **DB06701(3)** |
| **221** | **Dexpanthenol** | **DB09357(1)** |
| **222** | **Dextroamphetamine** | **DB01576(1)** |
| **223** | **Dextromethorphan** | **DB00514(7)** |
| **224** | **Dextrothyroxine** | **DB00509(2)** |
| 225 | D-glucose | DB01914(1) |
| **226** | **Diacerein** | **DB11994(1)** |
| **227** | **Diamorphine** | **DB01452(2)** |
| **228** | **Diazepam** | **DB00829(5)** |
| **229** | **Dichlorobenzyl alcohol** | **DB13269(1)** |
| **230** | **Diclofenac** | **CHEMBL139(5); DB00586(19)** |
| 231 | Didanosine | DB00900(4) |
| **232** | **Diethylcarbamazine** | **DB00711(1)** |
| **233** | **Diethylstilbestrol** | **CHEMBL411(2)** |
| **234** | **Diethyltoluamide** | **DB11282(2)** |
| **235** | **Diflunisal** | **CHEMBL898(2)** |
| **236** | **Difluprednate** | **DB06781(2)** |
| **237** | **Digoxin** | **DB00390(2)** |
| **238** | **Dihydroergocristine** | **DB13345(1)** |
| **239** | **Diltiazem** | **DB00343(6)** |
| 240 | Dimethyl sulfoxide | DB01093(2) |
| **241** | **Diphenhydramine** | **DB01075(5)** |
| 242 | Diphenidol | CHEMBL3544567(6) |
| 243 | Diroximel fumarate | DB14783(7) |
| **244** | **Disopyramide** | **CHEMBL517(1); DB00280(1)** |
| **245** | **Docetaxel** | **CHEMBL92(4); DB01248(1)** |
| **246** | **Doconexent** | **DB03756(1)** |
| **247** | **Dolutegravir** | **DB08930(5)** |
| **248** | **Domperidone** | **DB01184(1)** |
| **249** | **Donepezil** | **DB00843(7)** |
| **250** | **Dopamine** | **DB00988(5)** |
| **251** | **Doravirine** | **DB12301(1)** |
| **252** | **Doripenem** | **DB06211(1)** |
| **253** | **Dorzolamide** | **DB00869(1)** |
| **254** | **Dosulepin** | **DB09167(3)** |
| **255** | **Doxazosin** | **CHEMBL707(6); DB00590(2)** |
| **256** | **Doxepin** | **DB01142(10)** |
| **257** | **Doxorubicin** | **CHEMBL53463(6); DB00997(5)** |
| **258** | **Dronabinol** | **DB00470(13)** |
| **259** | **Dronedarone** | **DB04855(8)** |
| **260** | **Drospirenone** | **DB01395(1)** |
| **261** | **Droxicam** | **DB09215(1)** |
| **262** | **Duloxetine** | **DB00476(8)** |
| **263** | **Dydrogesterone** | **DB00378(1)** |
| **264** | **E-7016** | **CHEMBL3527000(3)** |
| **265** | **Ebastine** | **DB11742(2)** |
| **266** | **Efavirenz** | **DB00625(1)** |
| 267 | Eidd-2801 | DB15661(1) |
| **268** | **Eletriptan** | **DB00216(2)** |
| **269** | **Emedastine** | **DB01084(2)** |
| **270** | **Empagliflozin** | **DB09038(3)** |
| **271** | **Emtricitabine** | **DB00879(4)** |
| **272** | **Enalapril** | **DB00584(1)** |
| **273** | **Encainide** | **DB01228(2)** |
| **274** | **Enflurane** | **DB00228(1)** |
| **275** | **Entrectinib** | **DB11986(6)** |
| **276** | **Enzacamene** | **DB11219(3)** |
| **277** | **Epinephrine** | **DB00668(4)** |
| **278** | **Epirubicin** | **CHEMBL417(7)** |
| **279** | **Eplerenone** | **DB00700(2)** |
| **280** | **Epoprostenol** | **DB01240(2)** |
| **281** | **Ergocalciferol** | **DB00153(5)** |
| **282** | **Erlotinib** | **DB00530(1)** |
| **283** | **Ertugliflozin** | **DB11827(9)** |
| **284** | **Erythromycin** | **CHEMBL532(3); DB00199(1)** |
| **285** | **Escitalopram** | **DB01175(3)** |
| 286 | Esketamine | DB11823(1) |
| **287** | **Eslicarbazepine acetate** | **DB09119(7)** |
| **288** | **Esmolol** | **DB00187(1)** |
| **289** | **Esomeprazole** | **DB00736(4)** |
| **290** | **Estazolam** | **DB01215(1)** |
| **291** | **Estradiol** | **DB00783(20)** |
| **292** | **Estradiol acetate** | **DB13952(1)** |
| **293** | **Estradiol benzoate** | **DB13953(1)** |
| **294** | **Estradiol cypionate** | **DB13954(1)** |
| **295** | **Estradiol dienanthate** | **DB13955(1)** |
| **296** | **Estradiol valerate** | **DB13956(1)** |
| **297** | **Estriol** | **DB04573(5)** |
| **298** | **Estrone** | **DB00655(14)** |
| **299** | **Estrone sulfate** | **DB04574(2)** |
| **300** | **Eszopiclone** | **DB00402(3)** |
| 301 | Ethanol | DB00898(2) |
| **302** | **Ethinyl estradiol** | **CHEMBL691(10)** |
| **303** | **Ethinylestradiol** | **DB00977(12)** |
| **304** | **Ethionamide** | **DB00609(1)** |
| **305** | **Ethosuximide** | **CHEMBL696(7)** |
| **306** | **Ethotoin** | **DB00754(1)** |
| **307** | **Etizolam** | **DB09166(1)** |
| **308** | **Etodolac** | **CHEMBL622(6); DB00749(3)** |
| **309** | **Etonogestrel** | **DB00294(2)** |
| **310** | **Etoperidone** | **DB09194(22)** |
| **311** | **Etoposide** | **DB00773(4)** |
| **312** | **Etoricoxib** | **DB01628(5)** |
| **313** | **Eucalyptol** | **DB03852(2)** |
| **314** | **Exemestane** | **CHEMBL1200374(2)** |
| **315** | **Ezetimibe** | **DB00973(4)** |
| **316** | **Famciclovir** | **DB00426(1)** |
| **317** | **Famotidine** | **CHEMBL902(1); DB00927(1)** |
| **318** | **Favipiravir** | **DB12466(2)** |
| **319** | **Felbamate** | **DB00949(8)** |
| **320** | **Felodipine** | **DB01023(1)** |
| 321 | Fenfluramine | DB00574(1) |
| **322** | **Fenofibrate** | **CHEMBL672(4); DB01039(4)** |
| **323** | **Fenoprofen** | **CHEMBL3544569(3); DB00573(2)** |
| **324** | **Fentanyl** | **DB00813(4)** |
| **325** | **Fenthion** | **DB11412(1)** |
| 326 | Ferric cation | DB13949(1) |
| 327 | Ferric maltol | DB15598(1) |
| **328** | **Fexofenadine** | **DB00950(1)** |
| **329** | **Fidaxomicin** | **DB08874(1)** |
| **330** | **Finasteride** | **DB01216(1)** |
| **331** | **Fingolimod** | **DB08868(1)** |
| **332** | **Flecainide** | **DB01195(2)** |
| 333 | Florbetapir (18F) | DB09149(3) |
| **334** | **Flosequinan** | **DB13228(1)** |
| **335** | **Floxuridine** | **CHEMBL917(4); DB00322(4)** |
| 336 | Fluconazole | DB00196(2) |
| **337** | **Fludrocortisone** | **DB00687(2)** |
| **338** | **Flunarizine** | **DB04841(3)** |
| **339** | **Fluorodopa (18F)** | **DB13848(1)** |
| **340** | **Fluorouracil** | **CHEMBL185(9)** |
| **341** | **Fluoxetine** | **DB00472(6)** |
| **342** | **Fluphenazine** | **DB00623(1)** |
| **343** | **Flurazepam** | **DB00690(2)** |
| **344** | **Flurbiprofen** | **DB00712(2)** |
| **345** | **Flutamide** | **DB00499(1)** |
| **346** | **Fluticasone furoate** | **DB08906(1)** |
| **347** | **Fluticasone propionate** | **DB00588(1)** |
| **348** | **Fluvastatin** | **DB01095(5)** |
| **349** | **Fluvoxamine** | **DB00176(2)** |
| **350** | **Folic acid** | **DB00158(5)** |
| **351** | **Fomepizole** | **CHEMBL1308(4); DB01213(2)** |
| **352** | **Formestane** | **DB08905(1)** |
| **353** | **Formoterol** | **DB00983(8)** |
| **354** | **Fosinopril** | **DB00492(1)** |
| **355** | **Fosphenytoin** | **DB01320(1)** |
| **356** | **Fospropofol** | **DB06716(4)** |
| **357** | **Fostamatinib** | **DB12010(6)** |
| **358** | **Frovatriptan** | **DB00998(4)** |
| **359** | **Furazolidone** | **DB00614(2)** |
| **360** | **Furosemide** | **DB00695(2)** |
| **361** | **Fusidic acid** | **DB02703(5)** |
| **362** | **Galactose** | **DB11735(4)** |
| **363** | **Galantamine** | **DB00674(2)** |
| **364** | **Gatifloxacin** | **DB01044(2)** |
| **365** | **Gavestinel** | **DB06741(3)** |
| **366** | **Gemcitabine** | **DB00441(4)** |
| **367** | **Gemfibrozil** | **DB01241(3)** |
| **368** | **Gemifloxacin** | **DB01155(1)** |
| **369** | **Genistein** | **DB01645(1)** |
| 370 | Gilteritinib | DB12141(3) |
| **371** | **Glasdegib** | **DB11978(9)** |
| **372** | **Gliclazide** | **DB01120(8)** |
| **373** | **Glimepiride** | **DB00222(2)** |
| **374** | **Glipizide** | **DB01067(3)** |
| **375** | **Glyburide** | **DB01016(5)** |
| 376 | Glycine | DB00145(3) |
| **377** | **Glycyrrhizic acid** | **DB13751(2)** |
| **378** | **Granisetron** | **DB00889(2)** |
| **379** | **Griseofulvin** | **DB00400(1)** |
| **380** | **Guaifenesin** | **DB00874(1)** |
| **381** | **Guanabenz** | **DB00629(1)** |
| **382** | **Guanfacine** | **DB01018(3)** |
| **383** | **Halofantrine** | **DB01218(1)** |
| **384** | **Haloperidol** | **CHEMBL3544514(12); DB00502(9)** |
| 385 | Halothane | DB01159(6) |
| **386** | **Harmaline** | **DB13875(1)** |
| **387** | **Harmine** | **DB07919(2)** |
| **388** | **Hesperetin** | **DB01094(1)** |
| **389** | **Hexobarbital** | **DB01355(6)** |
| **390** | **Hydralazine** | **DB01275(13)** |
| **391** | **Hydrocodone** | **DB00956(12)** |
| **392** | **Hydrocortisone** | **DB00741(10)** |
| 393 | Hydrogen peroxide | DB11091(2) |
| **394** | **Hydromorphone** | **DB00327(2)** |
| **395** | **Hydroxychloroquine** | **DB01611(3)** |
| **396** | **Hydroxycitronellal** | **DB14187(2)** |
| 397 | Hydroxyurea | CHEMBL467(1) |
| **398** | **Hydroxyzine** | **DB00557(1)** |
| **399** | **Ibrutinib** | **DB09053(9)** |
| **400** | **Ibuprofen** | **CHEMBL3544649(3); DB01050(5)** |
| **401** | **Icosapent ethyl** | **DB08887(1)** |
| **402** | **Idarubicin** | **CHEMBL1117(2)** |
| **403** | **Idebenone** | **DB09081(4)** |
| **404** | **Idelalisib** | **DB09054(1)** |
| **405** | **Ifosfamide** | **DB01181(12)** |
| **406** | **Imatinib** | **CHEMBL941(6); DB00619(5)** |
| **407** | **Imidacloprid** | **DB11421(1)** |
| **408** | **Imipramine** | **DB00458(9)** |
| 409 | Indacaterol | DB05039(1) |
| **410** | **Indapamide** | **DB00808(7)** |
| **411** | **Indinavir** | **DB00224(1)** |
| **412** | **Indiplon** | **DB12590(1)** |
| **413** | **Indomethacin** | **CHEMBL6(3); DB00328(4)** |
| **414** | **Inositol** | **DB13178(1)** |
| **415** | **Inositol nicotinate** | **DB08949(6)** |
| 416 | Inulin | DB00638(1) |
| 417 | Iobenguane sulfate I-123 | DB09546(2) |
| 418 | Iodoform | DB13813(1) |
| 419 | Ipratropium | DB00332(4) |
| **420** | **Irbesartan** | **DB01029(9)** |
| **421** | **Irinotecan** | **DB00762(2)** |
| **422** | **Isocarboxazid** | **DB01247(1)** |
| **423** | **Isoniazid** | **CHEMBL64(11)** |
| **424** | **Isopropyl myristate** | **DB13966(2)** |
| **425** | **Isosorbide mononitrate** | **DB01020(3)** |
| **426** | **Isotretinoin** | **DB00982(5)** |
| **427** | **Istradefylline** | **DB11757(6)** |
| **428** | **Itraconazole** | **DB01167(1)** |
| **429** | **Ivacaftor** | **DB08820(2)** |
| **430** | **Ketamine** | **CHEMBL1039(7); DB01221(7)** |
| **431** | **Ketobemidone** | **DB06738(7)** |
| **432** | **Ketoconazole** | **DB01026(19)** |
| **433** | **Ketoprofen** | **CHEMBL571(3); DB01009(1)** |
| **434** | **Ketorolac** | **DB00465(2)** |
| **435** | **Ketotifen** | **DB00920(3)** |
| **436** | **Labetalol** | **DB00598(10)** |
| **437** | **Lacosamide** | **DB06218(1)** |
| **438** | **Lactulose** | **DB00581(3)** |
| **439** | **Lamivudine** | **DB00709(5)** |
| **440** | **Lamotrigine** | **DB00555(1)** |
| **441** | **Lansoprazole** | **DB00448(2)** |
| **442** | **Lapatinib** | **CHEMBL554(8)** |
| **443** | **Laquinimod** | **DB06685(6)** |
| **444** | **Lasofoxifene** | **DB06202(2)** |
| **445** | **Latanoprost** | **DB00654(3)** |
| **446** | **Latanoprostene bunod** | **DB11660(8)** |
| **447** | **Lauric acid** | **DB03017(1)** |
| **448** | **Leflunomide** | **DB01097(1)** |
| **449** | **Letermovir** | **DB12070(1)** |
| **450** | **Letrozole** | **DB01006(3)** |
| 451 | Leucovorin | DB00650(1) |
| **452** | **Levacetylmethadol** | **DB01227(3)** |
| **453** | **Levamlodipine** | **DB09237(4)** |
| **454** | **Levetiracetam** | **DB01202(1)** |
| **455** | **Levocarnitine** | **DB00583(2)** |
| **456** | **Levocetirizine** | **DB06282(8)** |
| **457** | **Levodopa** | **DB01235(14)** |
| **458** | **Levofloxacin** | **DB01137(2)** |
| **459** | **Levoleucovorin** | **DB11596(1)** |
| **460** | **Levomenthol** | **DB00825(1)** |
| 461 | Levonorgestrel | DB00367(4) |
| **462** | **Levosimendan** | **DB00922(2)** |
| **463** | **Levothyroxine** | **DB00451(2)** |
| **464** | **L-glutamine** | **DB00130(5)** |
| **465** | **Licofelone** | **DB04725(5)** |
| **466** | **Lidocaine** | **DB00281(5)** |
| 467 | Linaclotide | DB08890(1) |
| **468** | **Linagliptin** | **DB08882(12)** |
| **469** | **Linezolid** | **DB00601(2)** |
| **470** | **Liothyronine** | **CHEMBL1236469(7); DB00279(4)** |
| **471** | **Lisdexamfetamine** | **DB01255(2)** |
| **472** | **Lisofylline** | **DB12406(2)** |
| **473** | **Lixivaptan** | **CHEMBL49429(7)** |
| **474** | **Lobeglitazone** | **DB09198(2)** |
| **475** | **Lofexidine** | **DB04948(3)** |
| **476** | **Loperamide** | **DB00836(1)** |
| **477** | **Lopinavir** | **DB01601(3)** |
| **478** | **Loratadine** | **DB00455(6)** |
| **479** | **Lorazepam** | **DB00186(1)** |
| **480** | **Lorcaserin** | **DB04871(2)** |
| **481** | **Lormetazepam** | **DB13872(1)** |
| **482** | **Lornoxicam** | **DB06725(1)** |
| **483** | **Losartan** | **DB00678(3)** |
| **484** | **Loteprednol etabonate** | **DB14596(2)** |
| **485** | **Lovastatin** | **CHEMBL503(7); DB00227(3)** |
| **486** | **Loxoprofen** | **DB09212(2)** |
| 487 | Lumateperone | DB06077(3) |
| **488** | **Lumefantrine** | **DB06708(1)** |
| **489** | **Lumiracoxib** | **DB01283(18)** |
| **490** | **Lurasidone** | **DB08815(2)** |
| **491** | **Malathion** | **DB00772(3)** |
| **492** | **Mannitol** | **DB00742(1)** |
| **493** | **Maprotiline** | **DB00934(3)** |
| **494** | **M-Chlorophenylpiperazine** | **DB12110(1)** |
| **495** | **Mebendazole** | **DB00643(1)** |
| **496** | **Meclizine** | **DB00737(2)** |
| **497** | **Medroxyprogesterone acetate** | **DB00603(5)** |
| **498** | **Mefenamic acid** | **DB00784(1)** |
| **499** | **Mefloquine** | **DB00358(1)** |
| **500** | **Melatonin** | **DB01065(10)** |
| **501** | **Meloxicam** | **DB00814(2)** |
| 502 | Melphalan | CHEMBL852(2) |
| 503 | Memantine | DB01043(3) |
| **504** | **Mephenytoin** | **DB00532(4)** |
| **505** | **Mequinol** | **DB09516(3)** |
| 506 | Mercaptopurine | DB01033(9) |
| **507** | **Mesalazine** | **DB00244(1)** |
| **508** | **Mestranol** | **DB01357(2)** |
| **509** | **Metamfetamine** | **DB01577(5)** |
| **510** | **Methadone** | **DB00333(2)** |
| **511** | **Methenamine** | **DB06799(2)** |
| **512** | **Methimazole** | **DB00763(9)** |
| **513** | **Methocarbamol** | **DB00423(2)** |
| **514** | **Methotrexate** | **CHEMBL34259(3); DB00563(1)** |
| **515** | **Methyl nicotinate** | **DB13882(2)** |
| **516** | **Methyl salicylate** | **DB09543(1)** |
| **517** | **Methyldopa** | **CHEMBL459(5); DB00968(4)** |
| **518** | **Methylphenidate** | **DB00422(1)** |
| **519** | **Methylphenobarbital** | **DB00849(1)** |
| **520** | **Methylprednisolone** | **DB00959(15)** |
| **521** | **Metoclopramide** | **DB01233(2)** |
| **522** | **Metoprolol** | **CHEMBL3544687(4); DB00264(3)** |
| **523** | **Metronidazole** | **CHEMBL137(2); DB00916(1)** |
| **524** | **Metyrapone** | **DB01011(1)** |
| **525** | **Mexiletine** | **DB00379(2)** |
| **526** | **Mianserin** | **DB06148(6)** |
| **527** | **Midazolam** | **DB00683(4)** |
| **528** | **Midodrine** | **DB00211(1)** |
| **529** | **Midostaurin** | **DB06595(2)** |
| **530** | **Mifepristone** | **DB00834(2)** |
| **531** | **Milnacipran** | **DB04896(3)** |
| 532 | Miltefosine | DB09031(1) |
| **533** | **Minaprine** | **DB00805(1)** |
| **534** | **Minocycline** | **DB01017(3)** |
| **535** | **Mirabegron** | **CHEMBL2095212(10)** |
| **536** | **Mirtazapine** | **DB00370(3)** |
| **537** | **Misoprostol** | **DB00929(4)** |
| **538** | **Mitoxantrone** | **CHEMBL58(2)** |
| **539** | **Moclobemide** | **DB01171(15)** |
| **540** | **Modafinil** | **CHEMBL1373(4); DB00745(1)** |
| **541** | **Moexipril** | **DB00691(1)** |
| **542** | **Molsidomine** | **DB09282(1)** |
| **543** | **Mometasone** | **DB00764(1)** |
| **544** | **Mometasone furoate** | **DB14512(6)** |
| **545** | **Montelukast** | **DB00471(11)** |
| **546** | **Morphine** | **DB00295(5)** |
| **547** | **Moxidectin** | **DB11431(2)** |
| **548** | **Moxisylyte** | **DB09205(6)** |
| **549** | **Mupirocin** | **DB00410(1)** |
| **550** | **Mycophenolate mofetil** | **DB00688(8)** |
| **551** | **Mycophenolic acid** | **DB01024(3)** |
| **552** | **Nabumetone** | **CHEMBL1070(9); DB00461(23)** |
| **553** | **Nalidixic acid** | **DB00779(1)** |
| **554** | **Nalmefene** | **DB06230(5)** |
| **555** | **Naltrexone** | **DB00704(1)** |
| **556** | **Naproxen** | **DB00788(5)** |
| **557** | **Nateglinide** | **DB00731(7)** |
| **558** | **Nebivolol** | **DB04861(1)** |
| **559** | **Nefazodone** | **DB01149(3)** |
| **560** | **Nelarabine** | **DB01280(2)** |
| **561** | **Nelfinavir** | **DB00220(2)** |
| 562 | Netarsudil | DB13931(1) |
| **563** | **Netupitant** | **DB09048(2)** |
| **564** | **Nevirapine** | **DB00238(13)** |
| **565** | **Niacin** | **DB00627(6)** |
| **566** | **Nicardipine** | **DB00622(2)** |
| **567** | **Nicergoline** | **DB00699(1)** |
| **568** | **Nicoboxil** | **DB12911(2)** |
| **569** | **Nicotinamide** | **CHEMBL1140(4)** |
| **570** | **Nicotine** | **CHEMBL3(24); DB00184(14)** |
| **571** | **Nifedipine** | **CHEMBL193(4); DB01115(3)** |
| **572** | **Nintedanib** | **DB09079(4)** |
| **573** | **Nisoldipine** | **CHEMBL1726(2)** |
| **574** | **Nitazoxanide** | **DB00507(2)** |
| **575** | **Nitrofurantoin** | **DB00698(1)** |
| **576** | **Nitroglycerin** | **DB00727(5)** |
| 577 | Nitroprusside | DB00325(1) |
| 578 | Nitrous acid | DB09112(3) |
| **579** | **Nizatidine** | **DB00585(1)** |
| **580** | **Norepinephrine** | **DB00368(2)** |
| **581** | **Norethindrone acetate** | **CHEMBL1201146(19)** |
| **582** | **Norethisterone** | **DB00717(9)** |
| **583** | **Norgestimate** | **DB00957(3)** |
| **584** | **Odanacatib** | **DB06670(7)** |
| **585** | **Olanzapine** | **CHEMBL715(7); DB00334(5)** |
| **586** | **Olodaterol** | **DB09080(1)** |
| **587** | **Olopatadine** | **DB00768(2)** |
| **588** | **Olsalazine** | **DB01250(1)** |
| **589** | **Omeprazole** | **DB00338(4)** |
| **590** | **Ondansetron** | **DB00904(3)** |
| **591** | **Orantinib** | **DB12072(3)** |
| **592** | **Orciprenaline** | **DB00816(1)** |
| **593** | **Ornithine** | **DB00129(2)** |
| **594** | **Orphenadrine** | **DB01173(2)** |
| 595 | Oseltamivir | DB00198(1) |
| 596 | Oxabolone cipionate | DB13185(3) |
| **597** | **Oxaprozin** | **CHEMBL1071(7)** |
| **598** | **Oxcarbazepine** | **DB00776(8)** |
| **599** | **Oxetacaine** | **DB12532(2)** |
| **600** | **Oxybutynin** | **CHEMBL1231(2); DB01062(2)** |
| **601** | **Oxycodone** | **DB00497(9)** |
| **602** | **Oxymetholone** | **CHEMBL1200585(10)** |
| **603** | **Oxymorphone** | **DB01192(8)** |
| **604** | **Oxyquinoline** | **DB11145(2)** |
| 605 | Ozanimod | DB12612(8) |
| **606** | **Paclitaxel** | **CHEMBL428647(4); DB01229(3)** |
| **607** | **Palbociclib** | **DB09073(14)** |
| 608 | Pantoprazole | DB00213(3) |
| **609** | **Paraldehyde** | **DB09117(4)** |
| **610** | **Paramethadione** | **DB00617(1)** |
| 611 | Paroxetine | CHEMBL3544658(4) |
| **612** | **Pefloxacin** | **DB00487(2)** |
| **613** | **Penicillamine** | **CHEMBL1430(3)** |
| **614** | **Pentaerythritol tetranitrate** | **DB06154(4)** |
| **615** | **Pentoxifylline** | **CHEMBL628(3)** |
| 616 | Perboric acid | DB13235(3) |
| **617** | **Perhexiline** | **DB01074(3)** |
| **618** | **Perindopril** | **DB00790(3)** |
| **619** | **Perospirone** | **DB08922(1)** |
| **620** | **Perphenazine** | **DB00850(2)** |
| **621** | **Phenacetin** | **DB03783(2)** |
| **622** | **Phenazopyridine** | **DB01438(7)** |
| **623** | **Phendimetrazine** | **DB01579(1)** |
| **624** | **Phenelzine** | **CHEMBL1044(6); DB00780(4)** |
| **625** | **Phenethyl isothiocyanate** | **CHEMBL151649(5)** |
| **626** | **Pheniramine** | **DB01620(2)** |
| **627** | **Phenobarbital** | **CHEMBL40(7); DB01174(3)** |
| **628** | **Phenol** | **DB03255(1)** |
| **629** | **Phenoxymethylpenicillin** | **DB00417(2)** |
| **630** | **Phenprocoumon** | **DB00946(4)** |
| **631** | **Phentermine** | **DB00191(3)** |
| **632** | **Phenylbutazone** | **CHEMBL101(6)** |
| **633** | **Phenylbutyric acid** | **DB06819(1)** |
| **634** | **Phenylephrine** | **DB00388(1)** |
| **635** | **Phenytoin** | **CHEMBL16(10); DB00252(7)** |
| **636** | **Pholcodine** | **DB09209(4)** |
| **637** | **Pidolic acid** | **DB03088(1)** |
| **638** | **Pioglitazone** | **DB01132(8)** |
| **639** | **Piperaquine** | **DB13941(6)** |
| **640** | **Piperazine** | **DB00592(2)** |
| **641** | **Piroxicam** | **CHEMBL527(1); DB00554(1)** |
| **642** | **Pitolisant** | **DB11642(2)** |
| 643 | Pizotifen | DB06153(1) |
| 644 | Potassium nitrate | DB11090(2) |
| **645** | **Prasterone** | **DB01708(2)** |
| **646** | **Prasugrel** | **CHEMBL1201772(5); DB06209(2)** |
| **647** | **Pravastatin** | **CHEMBL1144(8); DB00175(10)** |
| **648** | **Prazosin** | **DB00457(5)** |
| **649** | **Prednisolone** | **DB00860(6)** |
| **650** | **Prednisolone acetate** | **DB15566(1)** |
| **651** | **Prednisone** | **CHEMBL635(1); DB00635(18)** |
| **652** | **Pregabalin** | **DB00230(1)** |
| **653** | **Pregnenolone** | **DB02789(3)** |
| 654 | Pretomanid | DB05154(1) |
| 655 | Prezatide | DB11296(1) |
| **656** | **Primidone** | **DB00794(2)** |
| **657** | **Procainamide** | **DB01035(1)** |
| **658** | **Procaine** | **CHEMBL569(2); DB00721(2)** |
| **659** | **Procarbazine** | **DB01168(2)** |
| **660** | **Prochlorperazine** | **CHEMBL728(3); DB00433(3)** |
| **661** | **Progesterone** | **DB00396(10)** |
| **662** | **Proguanil** | **DB01131(2)** |
| **663** | **Promazine** | **CHEMBL564(5); DB00420(4)** |
| **664** | **Promethazine** | **CHEMBL643(2); DB01069(3)** |
| **665** | **Propacetamol** | **DB09288(2)** |
| **666** | **Propafenone** | **DB01182(6)** |
| **667** | **Propanoic acid** | **DB03766(3)** |
| **668** | **Propiolactone** | **DB09348(2)** |
| **669** | **Propofol** | **DB00818(5)** |
| **670** | **Propranolol** | **CHEMBL31888(4); DB00571(4)** |
| **671** | **Prucalopride** | **DB06480(7)** |
| **672** | **Pseudoephedrine** | **DB00852(1)** |
| **673** | **Pyrazinamide** | **DB00339(1)** |
| **674** | **Pyrilamine** | **CHEMBL511(5)** |
| **675** | **Quazepam** | **DB01589(1)** |
| **676** | **Quetiapine** | **DB01224(4)** |
| **677** | **Quinapril** | **DB00881(3)** |
| **678** | **Quinestrol** | **DB04575(1)** |
| **679** | **Quinidine** | **DB00908(2)** |
| **680** | **Quinine** | **DB00468(1)** |
| **681** | **Rabeprazole** | **DB01129(1)** |
| **682** | **Rac-Methadone** | **CHEMBL651(3)** |
| **683** | **Raloxifene** | **DB00481(3)** |
| **684** | **Ramipril** | **DB00178(3)** |
| **685** | **Ranitidine** | **DB00863(3)** |
| **686** | **Ranolazine** | **DB00243(14)** |
| 687 | Remdesivir | DB14761(4) |
| **688** | **Repaglinide** | **DB00912(2)** |
| **689** | **Resveratrol** | **CHEMBL165(2)** |
| **690** | **Revefenacin** | **DB11855(1)** |
| **691** | **Rheinanthrone** | **DB13175(2)** |
| **692** | **Rifabutin** | **CHEMBL444633(6); DB00615(3)** |
| **693** | **Rifampin** | **CHEMBL374478(2)** |
| **694** | **Rifamycin** | **DB11753(1)** |
| **695** | **Rilpivirine** | **DB08864(7)** |
| **696** | **Riluzole** | **DB00740(4)** |
| **697** | **Risperidone** | **DB00734(1)** |
| **698** | **Ritonavir** | **CHEMBL163(7); DB00503(4)** |
| **699** | **Rivoglitazone** | **CHEMBL3526905(22)** |
| **700** | **Rizatriptan** | **DB00953(1)** |
| 701 | Rocuronium | DB00728(1) |
| **702** | **Rofecoxib** | **DB00533(7)** |
| **703** | **Roflumilast** | **DB01656(1)** |
| **704** | **Rolapitant** | **DB09291(1)** |
| **705** | **Romidepsin** | **DB06176(1)** |
| **706** | **Ropinirole** | **DB00268(3)** |
| **707** | **Ropivacaine** | **DB00296(3)** |
| **708** | **Roquinimex** | **DB11366(6)** |
| **709** | **Rosiglitazone** | **DB00412(14)** |
| **710** | **Rosuvastatin** | **DB01098(2)** |
| **711** | **Rotigotine** | **DB05271(2)** |
| **712** | **Roxithromycin** | **DB00778(3)** |
| **713** | **Rufinamide** | **DB06201(1)** |
| **714** | **Sacubitril** | **DB09292(1)** |
| **715** | **Salbutamol** | **DB01001(1)** |
| **716** | **Salmeterol** | **DB00938(2)** |
| **717** | **Saquinavir** | **CHEMBL114(8); DB01232(4)** |
| **718** | **Saxagliptin** | **DB06335(1)** |
| **719** | **Sb-649868** | **CHEMBL1272307(12)** |
| **720** | **Segesterone acetate** | **DB14583(6)** |
| **721** | **Selegiline** | **DB01037(2)** |
| 722 | Selenious acid | DB11127(9) |
| **723** | **Selexipag** | **DB11362(2)** |
| **724** | **Selumetinib** | **DB11689(15)** |
| 725 | Semaglutide | DB13928(3) |
| **726** | **Sennosides** | **DB11365(3)** |
| **727** | **Seratrodast** | **DB06739(6)** |
| **728** | **Sertraline** | **DB01104(4)** |
| **729** | **Sevoflurane** | **DB01236(3)** |
| **730** | **Sibrafiban** | **CHEMBL435176(2)** |
| **731** | **Sildenafil** | **CHEMBL910(12); DB00203(2)** |
| **732** | **Silibinin** | **DB09298(1)** |
| **733** | **Simvastatin** | **CHEMBL1064(6); DB00641(4)** |
| 734 | Siponimod | DB12371(12) |
| **735** | **Sirolimus** | **CHEMBL413(4); DB00877(8)** |
| **736** | **Sitagliptin** | **DB01261(4)** |
| 737 | Sodium oxybate | DB09072(2) |
| **738** | **Sofosbuvir** | **DB08934(3)** |
| **739** | **Solifenacin** | **DB01591(4)** |
| **740** | **Sorafenib** | **DB00398(3)** |
| **741** | **Spirapril** | **DB01348(1)** |
| **742** | **Spironolactone** | **CHEMBL1393(8); DB00421(5)** |
| **743** | **Stanolone** | **DB02901(3)** |
| **744** | **Stavudine** | **CHEMBL991(2)** |
| **745** | **Sulfamethoxazole** | **DB01015(7)** |
| **746** | **Sulfinpyrazone** | **DB01138(2)** |
| 747 | Sulfisoxazole | CHEMBL453(1) |
| **748** | **Sulindac** | **DB00605(2)** |
| **749** | **Sumatriptan** | **CHEMBL3544580(2); DB00669(3)** |
| **750** | **Sunitinib** | **CHEMBL535(14)** |
| **751** | **Suprofen** | **DB00870(3)** |
| 752 | Suvorexant | DB09034(1) |
| **753** | **Tacrine** | **DB00382(4)** |
| **754** | **Tacrolimus** | **CHEMBL269732(8); DB00864(4)** |
| **755** | **Tafenoquine** | **DB06608(1)** |
| **756** | **Tafluprost** | **DB08819(1)** |
| **757** | **Tamoxifen** | **DB00675(17)** |
| **758** | **Tamsulosin** | **DB00706(11)** |
| **759** | **Tapentadol** | **DB06204(1)** |
| **760** | **Tarenflurbil** | **DB05289(1)** |
| 761 | Taurine | DB01956(2) |
| **762** | **Tazarotene** | **DB00799(1)** |
| 763 | Tazemetostat | DB12887(1) |
| 764 | Tazobactam | DB01606(1) |
| **765** | **Tedizolid phosphate** | **DB09042(1)** |
| **766** | **Tegafur** | **DB09256(2)** |
| 767 | Tegafur-uracil | DB09327(11) |
| **768** | **Tegaserod** | **DB01079(1)** |
| **769** | **Telaprevir** | **DB05521(1)** |
| 770 | Telavancin | DB06402(1) |
| **771** | **Temazepam** | **DB00231(2)** |
| **772** | **Temsirolimus** | **DB06287(1)** |
| 773 | Tenapanor | DB11761(1) |
| **774** | **Teniposide** | **DB00444(3)** |
| **775** | **Tenofovir** | **DB14126(1)** |
| **776** | **Tenofovir alafenamide** | **DB09299(2)** |
| **777** | **Tenofovir disoproxil** | **DB00300(2)** |
| **778** | **Tenoxicam** | **DB00469(1)** |
| **779** | **Terazosin** | **DB01162(4)** |
| **780** | **Terbinafine** | **DB00857(11)** |
| **781** | **Terfenadine** | **DB00342(1)** |
| **782** | **Testosterone** | **DB00624(18)** |
| **783** | **Testosterone cypionate** | **DB13943(1)** |
| **784** | **Testosterone enanthate** | **DB13944(1)** |
| **785** | **Testosterone propionate** | **DB01420(1)** |
| **786** | **Testosterone undecanoate** | **DB13946(1)** |
| **787** | **Tetrabenazine** | **DB04844(2)** |
| **788** | **Tetracaine** | **DB09085(2)** |
| **789** | **Tezacaftor** | **DB11712(4)** |
| **790** | **Thalidomide** | **DB01041(14)** |
| **791** | **Theophylline** | **DB00277(4)** |
| **792** | **Thiabendazole** | **DB00730(1)** |
| 793 | Thiocolchicoside | DB11582(2) |
| **794** | **Thioguanine** | **CHEMBL727(6)** |
| **795** | **Thioridazine** | **CHEMBL479(6); DB00679(3)** |
| **796** | **Thymol** | **DB02513(3)** |
| 797 | Tiagabine | DB00906(1) |
| **798** | **Ticlopidine** | **CHEMBL833(7); DB00208(12)** |
| **799** | **Tienilic acid** | **DB04831(3)** |
| 800 | Timolol | DB00373(1) |
| 801 | Tioguanine | DB00352(6) |
| **802** | **Tiopronin** | **CHEMBL1314(1); DB06823(1)** |
| 803 | Tiotropium | CHEMBL1900528(16); DB01409(2) |
| **804** | **Tipiracil** | **DB09343(1)** |
| 805 | Tizanidine | DB00697(2) |
| **806** | **Tolazamide** | **CHEMBL817(4)** |
| **807** | **Tolbutamide** | **DB01124(1)** |
| **808** | **Tolfenamic acid** | **DB09216(15)** |
| **809** | **Tolmetin** | **DB00500(1)** |
| **810** | **Tolterodine** | **DB01036(4)** |
| **811** | **Toluene** | **DB11558(3)** |
| **812** | **Topiramate** | **DB00273(4)** |
| **813** | **Topiroxostat** | **DB01685(2)** |
| **814** | **Torasemide** | **DB00214(5)** |
| **815** | **Toremifene** | **DB00539(1)** |
| **816** | **Trabectedin** | **DB05109(5)** |
| **817** | **Tramadol** | **DB00193(6)** |
| **818** | **Trandolapril** | **DB00519(2)** |
| **819** | **Travoprost** | **DB00287(1)** |
| **820** | **Trazodone** | **DB00656(4)** |
| **821** | **Tretinoin** | **DB00755(6)** |
| **822** | **Triamcinolone** | **DB00620(1)** |
| **823** | **Triamterene** | **CHEMBL585(2); DB00384(2)** |
| **824** | **Triazolam** | **DB00897(2)** |
| 825 | Trichloroethylene | DB13323(1) |
| 826 | Triclabendazole | DB12245(2) |
| **827** | **Triethylenetetramine** | **DB06824(2)** |
| **828** | **Trifluridine** | **DB00432(2)** |
| **829** | **Triflusal** | **DB08814(1)** |
| 830 | Trihexyphenidyl | CHEMBL1490(1) |
| **831** | **Trimebutine** | **DB09089(5)** |
| **832** | **Trimethadione** | **DB00347(1)** |
| **833** | **Trimethoprim** | **DB00440(6)** |
| **834** | **Trofosfamide** | **DB12902(3)** |
| **835** | **Trolamine** | **DB13747(1)** |
| **836** | **Trovafloxacin** | **DB00685(1)** |
| **837** | **Udenafil** | **DB06267(1)** |
| **838** | **Umifenovir** | **DB13609(24)** |
| **839** | **Uridine triacetate** | **DB09144(1)** |
| **840** | **Vadimezan** | **DB06235(1)** |
| 841 | Valaciclovir | DB00577(5) |
| **842** | **Valbenazine** | **DB11915(3)** |
| **843** | **Valdecoxib** | **DB00580(14)** |
| **844** | **Valproic acid** | **CHEMBL109(19); DB00313(13)** |
| **845** | **Valrubicin** | **DB00385(2)** |
| **846** | **Valsartan** | **DB00177(1)** |
| **847** | **Vandetanib** | **DB05294(1)** |
| 848 | Vanoxerine | DB03701(1) |
| **849** | **Vemurafenib** | **DB08881(3)** |
| 850 | Venetoclax | DB11581(1) |
| **851** | **Venlafaxine** | **DB00285(6)** |
| **852** | **Verapamil** | **DB00661(7)** |
| **853** | **Vernakalant** | **DB06217(1)** |
| **854** | **Vicriviroc** | **DB06652(6)** |
| **855** | **Vilanterol** | **DB09082(2)** |
| **856** | **Vinblastine** | **DB00570(1)** |
| **857** | **Vincristine** | **CHEMBL90555(2)** |
| 858 | Vindesine | DB00309(1) |
| **859** | **Vinflunine** | **DB11641(1)** |
| **860** | **Vinorelbine** | **DB00361(1)** |
| **861** | **Vitamin A** | **DB00162(1)** |
| **862** | **Vitamin E** | **DB00163(7)** |
| **863** | **Vorapaxar** | **CHEMBL493982(3); DB09030(2)** |
| **864** | **Voriconazole** | **DB00582(8)** |
| **865** | **Vorinostat** | **DB02546(1)** |
| **866** | **Vortioxetine** | **DB09068(4)** |
| **867** | **Warfarin** | **CHEMBL3638312(18); DB00682(21)** |
| **868** | **Xylose** | **DB09419(3)** |
| **869** | **Zafirlukast** | **DB00549(7)** |
| **870** | **Zalcitabine** | **CHEMBL853(5)** |
| **871** | **Zaleplon** | **DB00962(1)** |
| **872** | **Zaltoprofen** | **DB06737(4)** |
| **873** | **Zidovudine** | **DB00495(4)** |
| **874** | **Zileuton** | **DB00744(5)** |
| **875** | **Ziprasidone** | **DB00246(12)** |
| **876** | **Zolmitriptan** | **DB00315(4)** |
| **877** | **Zolpidem** | **DB00425(6)** |
| **878** | **Zonisamide** | **DB00909(2)** |
| **879** | **Zopiclone** | **DB01198(4)** |
| **880** | **Zotepine** | **DB09225(5)** |
