# Supplementary material for: Computer-Aided Estimation of Biological Activity Profiles of Drug-Like Compounds Taking into Account Their Metabolism in Human Body
Source: Int J Mol Sci. 2020 Oct 11;21(20):7492. doi: 10.3390/ijms21207492 (PMC7593915; doi:10.3390/ijms21207492)
Supplement: Supplementary file 1 [file ijms-21-07492-s001.zip › Filimonov_DA-et-al-Table_S2.docx]

**Table S2.** Lists of biological activities belonging to the category “Pharmacological Effects”. NA is the number of active compounds; IAP is an Invariant Accuracy of Prediction obtained in leave-one-out cross-validation.

| **Activity** | **NA** | **IAP, LOO CV** |
| --- | --- | --- |
| Acaricide | 53 | 0.9548 |
| Acute neurologic disorders treatment | 4839 | 0.9217 |
| Allergic rhinitis treatment | 510 | 0.9618 |
| Alzheimer's disease treatment | 2639 | 0.9020 |
| Amyotrophic lateral sclerosis treatment | 463 | 0.9406 |
| Analeptic | 255 | 0.9073 |
| Analgesic, non-opioid | 12876 | 0.8945 |
| Andropause treatment | 87 | 0.9677 |
| Anesthetic inhalation | 20 | 0.9899 |
| Antiacne | 1814 | 0.9719 |
| Antialcoholic | 169 | 0.9313 |
| Antiallergic | 9002 | 0.9044 |
| Antianemic | 388 | 0.9696 |
| Antianginal | 3206 | 0.9428 |
| Antianorexic | 102 | 0.9636 |
| Antiarrhythmic | 3149 | 0.9481 |
| Antiarthritic | 13767 | 0.8770 |
| Antiasthmatic | 8917 | 0.8977 |
| Antibiotic | 7429 | 0.9837 |
| Anticataract | 129 | 0.9803 |
| Anticholelithogenic | 64 | 0.9873 |
| Antidepressant | 11164 | 0.9261 |
| Antidiabetic | 21403 | 0.8906 |
| Antidiabetic (type 1) | 103 | 0.9323 |
| Antidiabetic (type 2) | 3182 | 0.9431 |
| Antidiabetic symptomatic | 1868 | 0.9353 |
| Antidiarrheal | 371 | 0.9484 |
| Antidote | 203 | 0.9533 |
| Antidote, heavy metal | 105 | 0.9937 |
| Antidote, mercury | 3 | 0.9907 |
| Antidote, organophosphates | 6 | 0.8743 |
| Antieczematic atopic | 431 | 0.9121 |
| Antiemetic | 1571 | 0.9701 |
| Antiemphysemic | 679 | 0.9839 |
| Antiepileptic | 2471 | 0.9400 |
| Antifungal | 5074 | 0.9350 |
| Antifungal (Aspergillus) | 192 | 0.9591 |
| Antifungal (Candida) | 350 | 0.9575 |
| Antifungal (Cryptococcus) | 48 | 0.9340 |
| Antifungal (Pneumocystis) | 15 | 0.9135 |
| Antifungal enhancer | 23 | 0.9980 |
| Antiglaucomic | 2378 | 0.9509 |
| Antihelmintic | 1090 | 0.9638 |
| Antihelmintic (Fasciola) | 12 | 0.9825 |
| Antihelmintic (Nematodes) | 188 | 0.9554 |
| Antihelmintic (Trematoda) | 61 | 0.9982 |
| Antihemorrhagic | 28 | 0.9802 |
| Antihypercholesterolemic | 313 | 0.9140 |
| Antihyperlipoproteinemic | 112 | 0.9474 |
| Antihypermotility | 82 | 0.9692 |
| Antihypertensive | 14904 | 0.9158 |
| Antihypotensive | 44 | 0.9319 |
| Antiinfertility, female | 177 | 0.9667 |
| Antiischemic | 3722 | 0.9354 |
| Antileprosy | 82 | 0.9400 |
| Antileukemic | 5109 | 0.9415 |
| Antimigraine | 2172 | 0.9522 |
| Antimutagenic | 40 | 0.9696 |
| Antimycobacterial | 2523 | 0.9359 |
| Antimycoplasmal | 135 | 0.9847 |
| Antineoplastic (bladder cancer) | 378 | 0.9088 |
| Antineoplastic (bone cancer) | 71 | 0.8896 |
| Antineoplastic (brain cancer) | 1008 | 0.9007 |
| Antineoplastic (breast cancer) | 5668 | 0.9270 |
| Antineoplastic (carcinoma) | 2237 | 0.9567 |
| Antineoplastic (cervical cancer) | 2062 | 0.9557 |
| Antineoplastic (colon cancer) | 5493 | 0.9517 |
| Antineoplastic (colorectal cancer) | 5740 | 0.9456 |
| Antineoplastic (endocrine cancer) | 236 | 0.8872 |
| Antineoplastic (gastric cancer) | 635 | 0.9299 |
| Antineoplastic (glioblastoma multiforme) | 354 | 0.9200 |
| Antineoplastic (glioma) | 529 | 0.9261 |
| Antineoplastic (insulinoma) | 44 | 0.9999 |
| Antineoplastic (liver cancer) | 412 | 0.9074 |
| Antineoplastic (lung cancer) | 3075 | 0.9392 |
| Antineoplastic (lymphocytic leukemia) | 2813 | 0.9648 |
| Antineoplastic (lymphoma) | 992 | 0.9430 |
| Antineoplastic (melanoma) | 1714 | 0.9387 |
| Antineoplastic (multiple myeloma) | 208 | 0.8850 |
| Antineoplastic (non-small cell lung cancer) | 965 | 0.9142 |
| Antineoplastic (ovarian cancer) | 2047 | 0.9501 |
| Antineoplastic (pancreatic cancer) | 360 | 0.8955 |
| Antineoplastic (renal cancer) | 547 | 0.9042 |
| Antineoplastic (sarcoma) | 331 | 0.9211 |
| Antineoplastic (small cell lung cancer) | 211 | 0.8787 |
| Antineoplastic (solid tumors) | 778 | 0.8817 |
| Antineoplastic (squamous cell carcinoma) | 1272 | 0.9583 |
| Antineoplastic (uterine cancer) | 139 | 0.9412 |
| Antineoplastic antimetabolite | 176 | 0.9484 |
| Antineoplastic enhancer | 1241 | 0.9519 |
| Antineoplastic, alkylator | 125 | 0.9732 |
| Antinephritic | 237 | 0.9357 |
| Antineurogenic pain | 1162 | 0.9208 |
| Antiosteoporotic | 4347 | 0.9351 |
| Antiparasitic | 1270 | 0.9551 |
| Antiparkinsonian | 4787 | 0.9099 |
| Antiprotozoal | 27514 | 0.8845 |
| Antiprotozoal (Amoeba) | 373 | 0.9624 |
| Antiprotozoal (Babesia) | 5 | 0.9759 |
| Antiprotozoal (Coccidial) | 257 | 0.9367 |
| Antiprotozoal (Histomonas) | 8 | 0.9986 |
| Antiprotozoal (Leishmania) | 1988 | 0.9505 |
| Antiprotozoal (Plasmodium) | 20423 | 0.8895 |
| Antiprotozoal (Toxoplasma) | 337 | 0.9640 |
| Antiprotozoal (Trichomonas) | 253 | 0.9596 |
| Antiprotozoal (Trypanosoma) | 5319 | 0.9289 |
| Antiprotozoal activity enhancer | 5 | 1.0000 |
| Antipruritic, allergic | 161 | 0.8631 |
| Antipruritic, non-allergic | 75 | 0.9419 |
| Antipsoriatic | 4646 | 0.9229 |
| Antipsychotic | 11079 | 0.9299 |
| Antipyretic | 471 | 0.9225 |
| Antirickettsial | 19 | 0.9489 |
| Antischistosomal | 51 | 0.9761 |
| Antiseptic | 209 | 0.9566 |
| Antismoking | 220 | 0.9736 |
| Antispirochetal | 36 | 0.9685 |
| Antithrombocytopenic | 231 | 0.9777 |
| Antitreponemal | 12 | 0.9402 |
| Antituberculosic | 1980 | 0.9381 |
| Antitussive | 362 | 0.9582 |
| Antiulcerative | 2251 | 0.9417 |
| Antiviral (Bovine viral diarrhea virus) | 313 | 0.9861 |
| Antiviral (HIV) | 20302 | 0.9531 |
| Antiviral (Hepatitis B) | 449 | 0.9695 |
| Antiviral (Hepatitis C) | 6807 | 0.9702 |
| Antiviral (Hepatitis) | 9512 | 0.9615 |
| Antiviral (Herpes) | 1346 | 0.9700 |
| Antiviral (Herpesvirus 3, Human) | 256 | 0.9898 |
| Antiviral (Influenza A) | 359 | 0.9665 |
| Antiviral (Influenza B) | 29 | 0.9536 |
| Antiviral (Influenza) | 496 | 0.9500 |
| Antiviral (Parainfluenza) | 5 | 0.9999 |
| Antiviral (Poxvirus) | 103 | 0.9649 |
| Antiviral (Rhinovirus) | 367 | 0.9776 |
| Antiviral (Trachoma) | 12 | 0.9904 |
| Anxiolytic | 9298 | 0.9236 |
| Atherosclerosis treatment | 4552 | 0.8997 |
| Attention deficit/hyperactivity disorder treatment | 676 | 0.9755 |
| Autoimmune disorders treatment | 8944 | 0.8822 |
| Bipolar disorder treatment | 198 | 0.9649 |
| Bone diseases treatment | 5237 | 0.9285 |
| Bulimia treatment | 103 | 0.9976 |
| CNS active muscle relaxant | 140 | 0.9718 |
| Cachexia treatment | 265 | 0.9675 |
| Cardioprotectant | 251 | 0.9006 |
| Cardiovascular analeptic | 19 | 0.9363 |
| Chemopreventive | 361 | 0.9490 |
| Chemoprotective | 212 | 0.9341 |
| Choleretic | 144 | 0.9467 |
| Cholesterol absorption inhibitor | 194 | 0.9765 |
| Chronic obstructive pulmonary disease treatment | 4727 | 0.9388 |
| Cocain dependency treatment | 235 | 0.9611 |
| Cognition disorders treatment | 11383 | 0.9057 |
| Contraceptive | 1112 | 0.9628 |
| Contraceptive female | 138 | 0.9492 |
| Contraceptive male | 225 | 0.9793 |
| Corneal wound healing stimulator | 17 | 0.9983 |
| Cystic fibrosis treatment | 622 | 0.9440 |
| Cytostatic | 332 | 0.9257 |
| Dependence treatment | 2076 | 0.9465 |
| Diabetic retinopathy treatment | 277 | 0.9371 |
| Dysmenorrhea treatment | 171 | 0.9794 |
| Dyspepsia treatment | 77 | 0.9278 |
| Endometrios treatment | 297 | 0.9752 |
| Erectile dysfunction treatment | 942 | 0.9680 |
| Female sexual dysfunction treatment | 129 | 0.9715 |
| Gastroesophageal reflux disease treatment | 228 | 0.9670 |
| Gaucher disease treatment | 52 | 0.9813 |
| Gout treatment | 238 | 0.9659 |
| Growth stimulant | 115 | 0.9622 |
| HDL-cholesterol increasing | 414 | 0.9781 |
| Hair growth stimulant | 659 | 0.9744 |
| Heart failure treatment | 1632 | 0.9467 |
| Hepatic disorders treatment | 1179 | 0.9304 |
| Huntington's disease treatment | 149 | 0.9605 |
| Hypercalcemia treatment | 44 | 0.9603 |
| Hypogonadism treatment | 126 | 0.9991 |
| Inflammatory Bowel disease treatment | 2987 | 0.9172 |
| Irritable Bowel syndrome treatment | 2127 | 0.9531 |
| Lipoprotein disorders treatment | 4667 | 0.9539 |
| Liver cirrhosis treatment | 53 | 0.9260 |
| Liver fibrosis treatment | 465 | 0.9615 |
| Macular degeneration treatment | 256 | 0.9405 |
| Male reproductive disfunction treatment | 1373 | 0.9618 |
| Menstruation disorders treatment | 245 | 0.9426 |
| Metabolic disease treatment | 314 | 0.9338 |
| Mood disorders treatment | 11470 | 0.9246 |
| Movement disorders treatment | 44 | 0.9327 |
| Multiple sclerosis treatment | 3001 | 0.9266 |
| Muscle relaxant | 488 | 0.9412 |
| Myocardial infarction treatment | 623 | 0.9539 |
| Myocardial ischemia treatment | 1732 | 0.9607 |
| Narcotic antagonist | 112 | 0.9771 |
| Neurodegenerative diseases treatment | 8018 | 0.8767 |
| Neuropathy treatment | 26 | 0.8958 |
| Neutropenia treatment | 54 | 0.9997 |
| Obsessive-compulsive disorder treatment | 71 | 0.9537 |
| Osteoarthritis treatment | 346 | 0.9550 |
| Paget's disease treatment | 44 | 0.9647 |
| Pancreatic disorders treatment | 400 | 0.9733 |
| Peristaltic stimulant | 623 | 0.9855 |
| Postcoital contraceptive | 55 | 0.9938 |
| Postmenopausal disorders treatment | 282 | 0.9898 |
| Premature ejaculation treatment | 112 | 0.9807 |
| Premenstrual syndrome treatment | 27 | 0.9664 |
| Prokinetic | 520 | 0.9799 |
| Proliferative diseases treatment | 290 | 0.9055 |
| Prostate cancer treatment | 2944 | 0.9341 |
| Prostate disorders treatment | 922 | 0.9460 |
| Prostatic (benign) hyperplasia treatment | 2150 | 0.9684 |
| Psychosexual dysfunction treatment | 63 | 0.9499 |
| Pulmonary hypertension treatment | 139 | 0.8973 |
| Radioprotector | 263 | 0.8994 |
| Radiosensitizer | 263 | 0.9064 |
| Renal disease treatment | 1788 | 0.9540 |
| Renal failure treatment | 822 | 0.9630 |
| Respiratory analeptic | 193 | 0.8957 |
| Restenosis treatment | 1526 | 0.9591 |
| Rheumatoid arthritis treatment | 1925 | 0.9078 |
| Rhinitis treatment | 1487 | 0.9634 |
| Sepsis treatment | 160 | 0.9442 |
| Septic shock treatment | 1366 | 0.9466 |
| Shock treatment | 69 | 0.9668 |
| Skeletal muscle relaxant | 294 | 0.9455 |
| Skin whitener | 78 | 0.9804 |
| Sleep disorders treatment | 2425 | 0.9611 |
| Stroke treatment | 5038 | 0.9341 |
| Systemic lupus erythematosus treatment | 512 | 0.9427 |
| Tocolytic | 142 | 0.9772 |
| Transplant rejection treatment | 1978 | 0.9483 |
| Urinary incontinence treatment | 2675 | 0.9594 |
| Urologic disorders treatment | 4059 | 0.9277 |
| Uterine stimulant | 45 | 0.9707 |
| Vascular (periferal) disease treatment | 695 | 0.9328 |
| Wound healing agent | 638 | 0.9470 |
| Acetylcholine release stimulant | 146 | 0.9663 |
| Analgesic | 16272 | 0.8825 |
| Analgesic, opioid | 1848 | 0.9825 |
| Anesthetic general | 182 | 0.9337 |
| Anesthetic local | 515 | 0.9703 |
| Angiogenesis inhibitor | 5175 | 0.9161 |
| Angiogenesis stimulant | 24 | 0.8830 |
| Anti-Helicobacter pylori | 510 | 0.9694 |
| Antiamyloidogenic | 284 | 0.9215 |
| Antibacterial | 20404 | 0.9054 |
| Anticoagulant | 4934 | 0.9588 |
| Anticonvulsant | 4347 | 0.9293 |
| Antiinflammatory | 9804 | 0.8691 |
| Antimetabolite | 752 | 0.9903 |
| Antimitotic | 3727 | 0.9462 |
| Antineoplastic alkaloid | 92 | 0.9739 |
| Antineoplastic antibiotic | 1697 | 0.9843 |
| Antiobesity | 12280 | 0.9139 |
| Antioxidant | 1305 | 0.9540 |
| Antipruritic | 460 | 0.9173 |
| Antithrombotic | 654 | 0.8913 |
| Apoptosis agonist | 1904 | 0.8841 |
| Apoptosis antagonist | 442 | 0.9281 |
| Bone formation stimulant | 972 | 0.9674 |
| Bronchodilator | 2699 | 0.9708 |
| Cardiotonic | 7459 | 0.9208 |
| Cell adhesion inhibitor | 888 | 0.9693 |
| Cell wall synthesis inhibitor | 531 | 0.9656 |
| Cholesterol synthesis inhibitor | 85 | 0.9649 |
| DNA intercalator | 601 | 0.9832 |
| DNA synthesis inhibitor | 306 | 0.9120 |
| Diuretic | 1199 | 0.9526 |
| Expectorant | 204 | 0.9665 |
| Gastric antisecretory | 1683 | 0.9734 |
| Hematopoietic | 165 | 0.9653 |
| Hepatoprotectant | 545 | 0.9457 |
| Hypolipemic | 9312 | 0.9397 |
| Immunomodulator | 2776 | 0.9143 |
| Immunostimulant | 1135 | 0.9290 |
| Immunosuppressant | 4252 | 0.8993 |
| Insulin sensitizer | 237 | 0.9734 |
| Keratolytic | 48 | 0.9652 |
| Leukotriene synthesis inhibitor | 819 | 0.9867 |
| Mucolytic | 164 | 0.9644 |
| Photosensitizer | 76 | 0.9537 |
| Platelet aggregation inhibitor | 11688 | 0.9410 |
| Platelet antagonist | 2010 | 0.9577 |
| Psychostimulant | 417 | 0.9432 |
| Spasmolytic | 1668 | 0.9222 |
| Thrombolytic | 392 | 0.9757 |
| Tumour necrosis factor alpha release inhibitor | 2463 | 0.9478 |
| Uric acid excretion stimulant | 250 | 0.9622 |
| Vasodilator | 1692 | 0.9254 |
| Vasodilator, coronary | 288 | 0.8844 |
| Vasodilator, peripheral | 225 | 0.8816 |
