# Supplementary material for: Computer-Aided Estimation of Biological Activity Profiles of Drug-Like Compounds Taking into Account Their Metabolism in Human Body
Source: Int J Mol Sci. 2020 Oct 11;21(20):7492. doi: 10.3390/ijms21207492 (PMC7593915; doi:10.3390/ijms21207492)
Supplement: Supplementary file 1 [file ijms-21-07492-s001.zip › Filimonov_DA-et-al-Table_S3.docx]

**Table S3.** Lists of biological activities belonging to the category “Mechanisms of Action”. NA is the number of active compounds; IAP is an Invariant Accuracy of Prediction obtained in leave-one-out cross-validation.

| **Activity** | **NA** | **IAP. LOO CV** |
| --- | --- | --- |
| 1-Deoxy-D-xylulose-5-phosphate reductoisomerase inhibitor | 68 | 0.9997 |
| 11-Beta-hydroxysteroid dehydrogenase 1 inhibitor | 2713 | 0.9881 |
| 11-Beta-hydroxysteroid dehydrogenase 2 inhibitor | 299 | 0.9966 |
| 2,3-Oxidosqualene-lanosterol cyclase inhibitor | 304 | 0.9918 |
| 3 Beta-hydroxy-delta 5-steroid dehydrogenase inhibitor | 101 | 0.9932 |
| 4-Hydroxyphenylpyruvate dioxygenase inhibitor | 45 | 0.9985 |
| 5 Hydroxytryptamine 1 agonist | 2629 | 0.9897 |
| 5 Hydroxytryptamine 1 antagonist | 5526 | 0.986 |
| 5 Hydroxytryptamine 1A agonist | 1246 | 0.9923 |
| 5 Hydroxytryptamine 1A antagonist | 4233 | 0.9881 |
| 5 Hydroxytryptamine 1B agonist | 114 | 0.9941 |
| 5 Hydroxytryptamine 1B antagonist | 1161 | 0.9881 |
| 5 Hydroxytryptamine 1D agonist | 796 | 0.9965 |
| 5 Hydroxytryptamine 1D antagonist | 1173 | 0.9887 |
| 5 Hydroxytryptamine 1F agonist | 133 | 0.9995 |
| 5 Hydroxytryptamine 2 agonist | 1719 | 0.9944 |
| 5 Hydroxytryptamine 2 antagonist | 4691 | 0.9755 |
| 5 Hydroxytryptamine 2A agonist | 196 | 0.985 |
| 5 Hydroxytryptamine 2A antagonist | 3164 | 0.9845 |
| 5 Hydroxytryptamine 2B agonist | 112 | 0.9848 |
| 5 Hydroxytryptamine 2B antagonist | 1036 | 0.9793 |
| 5 Hydroxytryptamine 2C agonist | 656 | 0.9953 |
| 5 Hydroxytryptamine 2C antagonist | 1938 | 0.9874 |
| 5 Hydroxytryptamine 3 agonist | 114 | 0.9844 |
| 5 Hydroxytryptamine 3 antagonist | 1808 | 0.9872 |
| 5 Hydroxytryptamine 3A antagonist | 310 | 0.9719 |
| 5 Hydroxytryptamine 4 agonist | 525 | 0.9947 |
| 5 Hydroxytryptamine 4 antagonist | 618 | 0.9954 |
| 5 Hydroxytryptamine 6 agonist | 154 | 0.9959 |
| 5 Hydroxytryptamine 6 antagonist | 1377 | 0.9937 |
| 5 Hydroxytryptamine 7 antagonist | 726 | 0.987 |
| 5 Hydroxytryptamine agonist | 5161 | 0.9855 |
| 5 Hydroxytryptamine antagonist | 12847 | 0.9718 |
| 5 Hydroxytryptamine uptake inhibitor | 5503 | 0.988 |
| 5 Lipoxygenase activating protein inhibitor | 269 | 0.9953 |
| 5-Alpha-reductase 1 inhibitor | 563 | 0.9964 |
| 5-Alpha-reductase inhibitor | 1595 | 0.996 |
| 5-Lipoxygenase inhibitor | 3089 | 0.9782 |
| ABCA1 expression enhancer | 241 | 0.958 |
| Abl kinase inhibitor | 2510 | 0.9684 |
| Acetyl-CoA carboxylase 1 inhibitor | 319 | 0.9974 |
| Acetyl-CoA carboxylase 2 inhibitor | 441 | 0.9995 |
| Acetyl-CoA carboxylase inhibitor | 475 | 0.9922 |
| Acetyl-CoA transferase 1 inhibitor | 1660 | 0.9942 |
| Acetyl-CoA transferase 2 inhibitor | 331 | 0.9819 |
| Acetyl-CoA transferase inhibitor | 3149 | 0.9909 |
| Acetylcholine agonist | 3316 | 0.9764 |
| Acetylcholine antagonist | 5951 | 0.9615 |
| Acetylcholine M1 receptor agonist | 1647 | 0.9928 |
| Acetylcholine M1 receptor antagonist | 2149 | 0.9524 |
| Acetylcholine M2 receptor agonist | 204 | 0.986 |
| Acetylcholine M2 receptor antagonist | 2064 | 0.9902 |
| Acetylcholine M3 receptor agonist | 121 | 0.9838 |
| Acetylcholine M3 receptor antagonist | 2197 | 0.9904 |
| Acetylcholine M4 receptor agonist | 286 | 0.9968 |
| Acetylcholine M4 receptor antagonist | 789 | 0.9876 |
| Acetylcholine M5 receptor antagonist | 953 | 0.9923 |
| Acetylcholine muscarinic agonist | 1978 | 0.9897 |
| Acetylcholine muscarinic antagonist | 4503 | 0.963 |
| Acetylcholine nicotinic agonist | 1302 | 0.9806 |
| Acetylcholine nicotinic antagonist | 1520 | 0.9788 |
| Acetylcholine release stimulant | 146 | 0.9663 |
| Acetylcholinesterase inhibitor | 3713 | 0.9719 |
| Aconitate hydratase inhibitor | 22 | 0.9933 |
| Acyl-CoA dehydrogenase inhibitor | 15 | 0.9953 |
| Adenine nucleotide translocase inhibitor | 23 | 0.9685 |
| Adenosine A1 receptor agonist | 539 | 0.9976 |
| Adenosine A1 receptor antagonist | 3645 | 0.9872 |
| Adenosine A2 receptor agonist | 572 | 0.9985 |
| Adenosine A2 receptor antagonist | 4424 | 0.9865 |
| Adenosine A2a receptor agonist | 261 | 0.9993 |
| Adenosine A2a receptor antagonist | 2851 | 0.987 |
| Adenosine A2b receptor agonist | 17 | 0.9993 |
| Adenosine A2b receptor antagonist | 1247 | 0.9908 |
| Adenosine A3 receptor agonist | 181 | 0.9952 |
| Adenosine A3 receptor antagonist | 1756 | 0.9795 |
| Adenosine deaminase inhibitor | 249 | 0.9909 |
| Adenosine kinase inhibitor | 658 | 0.9973 |
| Adenosine uptake inhibitor | 57 | 0.9858 |
| Adenylate cyclase I inhibitor | 54 | 0.998 |
| Adenylate cyclase I stimulant | 43 | 1 |
| Adenylate cyclase inhibitor | 207 | 0.9503 |
| Adenylate cyclase stimulant | 91 | 0.9505 |
| Adenylate kinase inhibitor | 26 | 0.9456 |
| Adrenaline agonist | 6453 | 0.9745 |
| Adrenaline antagonist | 7076 | 0.9754 |
| Adrenaline uptake inhibitor | 3215 | 0.9901 |
| AICAR transformylase inhibitor | 60 | 0.9823 |
| Alcohol dehydrogenase inhibitor | 151 | 0.9664 |
| Alcohol oxidase inhibitor | 17 | 0.9919 |
| Aldehyde dehydrogenase inhibitor | 124 | 0.9644 |
| Aldehyde oxidase inhibitor | 29 | 0.9198 |
| Aldose reductase inhibitor | 2138 | 0.9927 |
| Aldosterone antagonist | 226 | 0.9839 |
| Alkaline phosphatase inhibitor | 419 | 0.9496 |
| Alkylator | 742 | 0.9654 |
| Alpha 1 adrenoreceptor agonist | 1000 | 0.9849 |
| Alpha 1 adrenoreceptor antagonist | 4136 | 0.9818 |
| Alpha 1a adrenoreceptor agonist | 140 | 0.9995 |
| Alpha 1a adrenoreceptor antagonist | 3345 | 0.9859 |
| Alpha 1b adrenoreceptor antagonist | 1449 | 0.9873 |
| Alpha 1d adrenoreceptor antagonist | 1639 | 0.989 |
| Alpha 1L adrenoreceptor agonist | 3 | 0.9945 |
| Alpha 1L adrenoreceptor antagonist | 11 | 0.9725 |
| Alpha 2 adrenoreceptor agonist | 916 | 0.9855 |
| Alpha 2 adrenoreceptor antagonist | 1780 | 0.9828 |
| Alpha 2a adrenoreceptor agonist | 24 | 0.997 |
| Alpha 2a adrenoreceptor antagonist | 724 | 0.9827 |
| Alpha 2b adrenoreceptor agonist | 86 | 0.9905 |
| Alpha 2b adrenoreceptor antagonist | 463 | 0.9807 |
| Alpha 2c adrenoreceptor agonist | 91 | 0.9985 |
| Alpha 2c adrenoreceptor antagonist | 564 | 0.9824 |
| Alpha 2d adrenoreceptor agonist | 44 | 0.999 |
| Alpha 2d adrenoreceptor antagonist | 94 | 0.9905 |
| Alpha adrenoreceptor agonist | 1697 | 0.9821 |
| Alpha adrenoreceptor antagonist | 5519 | 0.9759 |
| Alpha-mannosidase inhibitor | 64 | 0.9881 |
| Alpha-N-acetylglucosaminidase inhibitor | 4 | 0.9861 |
| Amidase inhibitor | 130 | 0.9711 |
| Aminoacyl-tRNA synthetase inhibitor | 549 | 0.9753 |
| Aminopeptidase A inhibitor | 67 | 0.9995 |
| Aminopeptidase B inhibitor | 58 | 0.9612 |
| Aminopeptidase I inhibitor | 26 | 0.988 |
| Aminopeptidase microsomal inhibitor | 508 | 0.9872 |
| Aminopeptidase N inhibitor | 481 | 0.989 |
| Aminopeptidase P inhibitor | 10 | 1 |
| AMPA receptor agonist | 167 | 0.997 |
| AMPA receptor antagonist | 1971 | 0.9917 |
| Amyloid beta aggregation inhibitor | 39 | 0.9788 |
| Amyloid beta precursor protein antagonist | 426 | 0.9383 |
| Analgesic | 16272 | 0.8825 |
| Analgesic, opioid | 1848 | 0.9825 |
| Androgen agonist | 633 | 0.983 |
| Androgen antagonist | 1855 | 0.979 |
| Anesthetic general | 182 | 0.9337 |
| Anesthetic local | 515 | 0.9703 |
| Angiogenesis inhibitor | 5175 | 0.9161 |
| Angiogenesis stimulant | 24 | 0.883 |
| Angiotensin AT1 receptor antagonist | 5152 | 0.9921 |
| Angiotensin AT1A receptor antagonist | 1609 | 0.9982 |
| Angiotensin AT1B receptor antagonist | 663 | 0.9986 |
| Angiotensin AT2 receptor agonist | 7 | 0.9877 |
| Angiotensin AT2 receptor antagonist | 1120 | 0.998 |
| Angiotensin II receptor antagonist | 5830 | 0.9934 |
| Angiotensin-converting enzyme inhibitor | 1629 | 0.9917 |
| Anti-Helicobacter pylori | 510 | 0.9694 |
| Antiadrenergic | 7147 | 0.9743 |
| Antiamyloidogenic | 284 | 0.9215 |
| Antibacterial | 20404 | 0.9054 |
| Anticoagulant | 4934 | 0.9588 |
| Anticonvulsant | 4347 | 0.9293 |
| Antidiuretic hormone agonist | 316 | 0.9926 |
| Antidiuretic hormone antagonist | 1857 | 0.9941 |
| Antihistaminic | 6127 | 0.9692 |
| Antiinflammatory | 9804 | 0.8691 |
| Antimetabolite | 752 | 0.9903 |
| Antimitotic | 3727 | 0.9462 |
| Antineoplastic alkaloid | 92 | 0.9739 |
| Antineoplastic antibiotic | 1697 | 0.9843 |
| Antiobesity | 12280 | 0.9139 |
| Antioxidant | 1305 | 0.954 |
| Antipruritic | 460 | 0.9173 |
| Antithrombotic | 654 | 0.8913 |
| Apolipoprotein B-100 inhibitor | 92 | 0.9983 |
| Apoptosis agonist | 1904 | 0.8841 |
| Apoptosis antagonist | 442 | 0.9281 |
| Arginase inhibitor | 81 | 0.9973 |
| Argininosuccinate synthase inhibitor | 21 | 0.9927 |
| Aromatase inhibitor | 1901 | 0.9909 |
| Aryl hydrocarbon receptor agonist | 125 | 0.9114 |
| ATP citrate lysase inhibitor | 59 | 0.9992 |
| ATPase (Vacuolar H+) inhibitor | 78 | 0.9907 |
| ATPase inhibitor | 155 | 0.9394 |
| Aurora-A kinase inhibitor | 5492 | 0.9764 |
| Aurora-B kinase inhibitor | 3507 | 0.9695 |
| Aurora-C kinase inhibitor | 199 | 0.9689 |
| Baculoviral IAP repeat-containing protein 2 inhibitor | 115 | 0.9984 |
| Baculoviral IAP repeat-containing protein 3 inhibitor | 33 | 0.9974 |
| Baculoviral IAP repeat-containing protein 4 inhibitor | 214 | 0.9903 |
| Baculoviral IAP repeat-containing protein inhibitor | 362 | 0.9909 |
| Bcl-xL inhibitor | 253 | 0.9775 |
| Bcl2 antagonist | 315 | 0.9661 |
| Benzodiazepine agonist | 457 | 0.983 |
| Benzodiazepine antagonist | 520 | 0.9914 |
| Benzodiazepine inverse agonist | 60 | 0.9909 |
| Benzodiazepine receptor peripheral-type antagonist | 464 | 0.9966 |
| Beta 1 adrenoreceptor agonist | 778 | 0.9966 |
| Beta 1 adrenoreceptor antagonist | 872 | 0.9896 |
| Beta 2 adrenoreceptor agonist | 2308 | 0.9745 |
| Beta 2 adrenoreceptor antagonist | 884 | 0.991 |
| Beta 3 adrenoreceptor agonist | 2471 | 0.9984 |
| Beta 3 adrenoreceptor antagonist | 233 | 0.9909 |
| Beta adrenoreceptor agonist | 4781 | 0.9829 |
| Beta adrenoreceptor antagonist | 1693 | 0.9851 |
| Beta amyloid protein antagonist | 125 | 0.9791 |
| Beta lactamase inhibitor | 30509 | 0.8594 |
| Beta tubulin antagonist | 664 | 0.9933 |
| Biliverdin reductase inhibitor | 11 | 0.9959 |
| Bombesin agonist | 274 | 0.9989 |
| Bombesin antagonist | 171 | 0.9818 |
| Bone formation stimulant | 972 | 0.9674 |
| Bone morphogenic protein 1 inhibitor | 350 | 0.9999 |
| Bradykinin B1 receptor agonist | 8 | 1 |
| Bradykinin B1 receptor antagonist | 1311 | 0.9971 |
| Bradykinin B2 receptor agonist | 9 | 0.9999 |
| Bradykinin B2 receptor antagonist | 663 | 0.9981 |
| Bronchodilator | 2699 | 0.9708 |
| Butyrylcholinesterase inhibitor | 1734 | 0.9863 |
| c-Src kinase inhibitor | 3915 | 0.9734 |
| Ca2+-transporting ATPase inhibitor | 12 | 0.8723 |
| Calcineurin inhibitor | 13 | 0.9997 |
| Calcitonin gene-related peptide 1 receptor antagonist | 446 | 0.9963 |
| Calcitonin receptor agonist | 17 | 0.9628 |
| Calcium channel (voltage-sensitive) blocker | 5254 | 0.9652 |
| Calcium channel activator | 75 | 0.901 |
| Calcium channel blocker | 7478 | 0.9591 |
| Calcium channel L-type blocker | 2108 | 0.9873 |
| Calcium channel N-type blocker | 2655 | 0.9578 |
| Calcium channel T-type blocker | 1161 | 0.9901 |
| Calcium release-activated channel blocker | 690 | 0.9956 |
| Calcium-dependent phospholipase A2 inhibitor | 99 | 0.9997 |
| Calcium-independent phospholipase A2 inhibitor | 63 | 0.9995 |
| Calcium-sensing receptor agonist | 140 | 0.9953 |
| Calcium-sensing receptor antagonist | 319 | 0.9969 |
| Calpain inhibitor | 1353 | 0.9107 |
| Cannabinoid CB1 receptor agonist | 1900 | 0.9896 |
| Cannabinoid CB1 receptor antagonist | 2616 | 0.9831 |
| Cannabinoid CB2 receptor agonist | 2170 | 0.9905 |
| Cannabinoid CB2 receptor antagonist | 1223 | 0.9916 |
| Cannabinoid receptor agonist | 3591 | 0.9883 |
| Carbamoyl-phosphate synthase (ammonia) inhibitor | 15 | 0.9942 |
| Carbonic anhydrase I inhibitor | 2450 | 0.9929 |
| Carbonic anhydrase I stimulant | 56 | 0.9997 |
| Carbonic anhydrase II inhibitor | 3148 | 0.9928 |
| Carbonic anhydrase II stimulant | 59 | 0.9946 |
| Carbonic anhydrase III inhibitor | 87 | 0.9577 |
| Carbonic anhydrase inhibitor | 3698 | 0.9919 |
| Carbonic anhydrase IV inhibitor | 1107 | 0.9936 |
| Carbonic anhydrase IV stimulant | 50 | 0.9999 |
| Carbonic anhydrase IX inhibitor | 1591 | 0.9933 |
| Carbonic anhydrase stimulant | 74 | 0.992 |
| Carbonic anhydrase V inhibitor | 258 | 0.9879 |
| Carbonic anhydrase VI inhibitor | 162 | 0.9769 |
| Carbonic anhydrase VII inhibitor | 305 | 0.9871 |
| Carbonic anhydrase XII inhibitor | 1140 | 0.9933 |
| Carbonic anhydrase XIII inhibitor | 142 | 0.9763 |
| Carbonic anhydrase XIV inhibitor | 403 | 0.9903 |
| Carbonic anhydrase XV inhibitor | 80 | 0.9637 |
| Carboxypeptidase B inhibitor | 108 | 0.986 |
| Cardiotonic | 7459 | 0.9208 |
| Caspase 2 inhibitor | 32 | 0.9987 |
| Caspase 3 inhibitor | 1112 | 0.9663 |
| Caspase 6 inhibitor | 147 | 0.999 |
| Caspase 7 inhibitor | 937 | 0.9708 |
| Catalase inhibitor | 69 | 0.9886 |
| Catechol O methyltransferase inhibitor | 199 | 0.9923 |
| Cathepsin B inhibitor | 1187 | 0.9884 |
| Cathepsin D inhibitor | 1095 | 0.9843 |
| Cathepsin K inhibitor | 1703 | 0.9952 |
| Cathepsin L inhibitor | 1386 | 0.9869 |
| Cathepsin S inhibitor | 1675 | 0.9968 |
| CC chemokine 1 receptor antagonist | 400 | 0.9925 |
| CC chemokine 10 receptor antagonist | 10 | 1 |
| CC chemokine 2 receptor antagonist | 903 | 0.9922 |
| CC chemokine 3 receptor antagonist | 666 | 0.9924 |
| CC chemokine 4 receptor antagonist | 190 | 0.9794 |
| CC chemokine 5 receptor antagonist | 1283 | 0.9932 |
| CC chemokine 8 receptor antagonist | 100 | 0.9923 |
| CC chemokine 9 receptor antagonist | 24 | 0.9751 |
| CC chemokine receptor antagonist | 4810 | 0.9837 |
| CD80 antagonist | 81 | 1 |
| CDK/Cyclin complex inhibitor | 11122 | 0.9788 |
| CDK1/cyclin B inhibitor | 3641 | 0.9869 |
| CDK2/cyclin A inhibitor | 3835 | 0.9784 |
| CDK2/cyclin A3 inhibitor | 318 | 0.9972 |
| CDK2/cyclin E inhibitor | 3750 | 0.9903 |
| CDK2/cyclin E2 inhibitor | 330 | 0.9748 |
| CDK3/cyclin E inhibitor | 101 | 0.9625 |
| CDK4/cyclin D inhibitor | 4521 | 0.9919 |
| CDK4/cyclin D1 inhibitor | 3269 | 0.9923 |
| CDK4/cyclin D3 inhibitor | 497 | 0.9997 |
| CDK6/cyclin D3 inhibitor | 715 | 0.9938 |
| CDK7/cyclin H inhibitor | 334 | 0.986 |
| CDK9/cyclin T1 inhibitor | 792 | 0.9897 |
| Cell adhesion inhibitor | 888 | 0.9693 |
| Cell wall synthesis inhibitor | 531 | 0.9656 |
| Ceramide glucosyltransferase inhibitor | 65 | 0.9792 |
| Check point kinase 1 inhibitor | 2840 | 0.982 |
| Check point kinase 2 inhibitor | 781 | 0.9748 |
| Cholecystokinin A agonist | 255 | 0.9963 |
| Cholecystokinin A antagonist | 1668 | 0.9946 |
| Cholecystokinin agonist | 285 | 0.9964 |
| Cholecystokinin antagonist | 2688 | 0.9941 |
| Cholecystokinin B agonist | 29 | 0.99 |
| Cholecystokinin B antagonist | 1948 | 0.9962 |
| Cholesterol ester transfer protein antagonist | 931 | 0.996 |
| Cholesterol esterase inhibitor | 295 | 0.9945 |
| Cholesterol synthesis inhibitor | 85 | 0.9649 |
| Cholinergic antagonist | 6110 | 0.9602 |
| Chymotrypsin inhibitor | 377 | 0.9694 |
| Collagenase 3 inhibitor | 2676 | 0.9862 |
| Collagenase inhibitor | 1082 | 0.9907 |
| Corticosteroid-like | 126 | 0.9994 |
| Corticotropin releasing factor 1 receptor antagonist | 1191 | 0.9985 |
| Creatine kinase inhibitor | 30 | 0.9791 |
| CXC chemokine 1 receptor antagonist | 134 | 0.979 |
| CXC chemokine 2 receptor antagonist | 185 | 0.9892 |
| CXC chemokine 3 receptor antagonist | 450 | 0.9975 |
| CXC chemokine 4 receptor antagonist | 225 | 0.993 |
| CXC chemokine 5 receptor antagonist | 332 | 0.9943 |
| CXC chemokine receptor agonist | 141 | 0.9994 |
| CXC chemokine receptor antagonist | 2177 | 0.9881 |
| Cyclic AMP phosphodiesterase inhibitor | 230 | 0.9128 |
| Cyclin-dependent kinase 1 inhibitor | 1816 | 0.9735 |
| Cyclin-dependent kinase 2 inhibitor | 3524 | 0.9733 |
| Cyclin-dependent kinase 3 inhibitor | 25 | 0.9572 |
| Cyclin-dependent kinase 4 inhibitor | 1239 | 0.9894 |
| Cyclin-dependent kinase 5 inhibitor | 1224 | 0.9694 |
| Cyclin-dependent kinase 6 inhibitor | 80 | 0.979 |
| Cyclin-dependent kinase 7 inhibitor | 257 | 0.9338 |
| Cyclin-dependent kinase inhibitor | 6299 | 0.9649 |
| Cyclooxygenase 1 inhibitor | 2126 | 0.9731 |
| Cyclooxygenase 2 inhibitor | 4879 | 0.9751 |
| Cyclooxygenase inhibitor | 6529 | 0.966 |
| Cystathionine beta-synthase inhibitor | 12 | 0.992 |
| Cytokine production inhibitor | 758 | 0.9609 |
| Cytokine production stimulant | 260 | 0.9871 |
| D-Ala-D-Ala ligase inhibitor | 6 | 0.971 |
| Diacylglycerol O-acyltransferase inhibitor | 659 | 0.9902 |
| Diamine oxidase inhibitor | 135 | 0.9946 |
| Dihydrofolate reductase inhibitor | 2608 | 0.9926 |
| Dihydroorotase inhibitor | 64 | 0.9944 |
| Dihydroorotate dehydrogenase inhibitor | 483 | 0.9905 |
| Dihydroorotate oxidase inhibitor | 84 | 0.9871 |
| Dihydropteroate synthase inhibitor | 57 | 0.9961 |
| Dipeptidyl peptidase I inhibitor | 124 | 0.9876 |
| Dipeptidyl peptidase II inhibitor | 429 | 0.9918 |
| Dipeptidyl peptidase inhibitor | 3697 | 0.992 |
| Dipeptidyl peptidase IV inhibitor | 3423 | 0.9938 |
| Dipeptidyl peptidase IX inhibitor | 346 | 0.999 |
| Dipeptidyl peptidase VIII inhibitor | 430 | 0.9989 |
| Diuretic | 1199 | 0.9526 |
| DNA damaging | 476 | 0.97 |
| DNA directed RNA polymerase inhibitor | 1365 | 0.9628 |
| DNA gyrase inhibitor | 527 | 0.988 |
| DNA intercalator | 601 | 0.9832 |
| DNA methylase inhibitor | 224 | 0.9252 |
| DNA repair enzyme inhibitor | 175 | 0.9708 |
| DNA synthesis inhibitor | 306 | 0.912 |
| DNA topoisomerase III inhibitor | 16 | 0.9999 |
| DNA topoisomerase IV inhibitor | 183 | 0.9908 |
| DNA-dependent protein kinase inhibitor | 779 | 0.9919 |
| DOPA decarboxylase inhibitor | 12 | 0.954 |
| Dopamine autoreceptor agonist | 161 | 0.9988 |
| Dopamine beta hydroxylase inhibitor | 155 | 0.9825 |
| Dopamine D1 agonist | 313 | 0.9982 |
| Dopamine D1 antagonist | 1128 | 0.9876 |
| Dopamine D2 agonist | 528 | 0.9936 |
| Dopamine D2 antagonist | 5530 | 0.9849 |
| Dopamine D3 agonist | 248 | 0.9936 |
| Dopamine D3 antagonist | 2182 | 0.9869 |
| Dopamine D4 agonist | 646 | 0.9896 |
| Dopamine D4 antagonist | 1449 | 0.9884 |
| Dopamine transporter inhibitor | 2244 | 0.9884 |
| Dopamine uptake inhibitor | 1907 | 0.9941 |
| Elastase inhibitor | 1295 | 0.9908 |
| Electron transport complex I inhibitor | 78 | 0.9943 |
| Endothelial nitric-oxide synthase inhibitor | 353 | 0.9962 |
| Endothelin A receptor antagonist | 2172 | 0.998 |
| Endothelin B receptor antagonist | 1442 | 0.9973 |
| Endothelin receptor antagonist | 2995 | 0.9966 |
| Endothelin-converting enzyme inhibitor | 525 | 0.9879 |
| Enoyl-[acyl-carrier-protein] reductase inhibitor | 242 | 0.9805 |
| Epidermal growth factor antagonist | 896 | 0.9897 |
| Epidermal growth factor receptor kinase inhibitor | 7810 | 0.9676 |
| Epithelial sodium channel blocker | 318 | 0.9957 |
| ErbB-2 antagonist | 2892 | 0.9791 |
| ErbB-4 antagonist | 179 | 0.966 |
| Erythropoietin receptor agonist | 10 | 0.9999 |
| Estrogen agonist | 1383 | 0.9885 |
| Estrogen antagonist | 2550 | 0.9575 |
| Estrogen beta receptor agonist | 440 | 0.9961 |
| Estrogen receptor alpha antagonist | 1547 | 0.97 |
| Estrogen receptor beta antagonist | 1792 | 0.9658 |
| Estrone sulfatase inhibitor | 374 | 0.9979 |
| Expectorant | 204 | 0.9665 |
| Factor VII inhibitor | 290 | 0.9968 |
| Factor VIIa inhibitor | 438 | 0.993 |
| Factor X inhibitor | 3526 | 0.994 |
| Factor Xa inhibitor | 2785 | 0.992 |
| Factor XIIIa inhibitor | 232 | 0.9907 |
| Farnesyltransferase inhibitor | 3468 | 0.9894 |
| Fatty acid synthase inhibitor | 129 | 0.9789 |
| Ferrochelatase inhibitor | 5 | 0.9926 |
| Fibroblast growth factor 1 antagonist | 1845 | 0.9708 |
| Fibroblast growth factor 2 antagonist | 677 | 0.9791 |
| Fibroblast growth factor 3 antagonist | 1117 | 0.9807 |
| Follicle-stimulating hormone agonist | 716 | 0.9969 |
| Follicle-stimulating hormone antagonist | 87 | 0.9997 |
| Fructose-1,6-bisphosphatase inhibitor | 418 | 0.9981 |
| Fumarate hydratase inhibitor | 24 | 0.9974 |
| GABA A receptor agonist | 745 | 0.9771 |
| GABA A receptor antagonist | 3146 | 0.9893 |
| GABA aminotransferase inhibitor | 43 | 0.9678 |
| GABA B receptor agonist | 23 | 0.9802 |
| GABA B receptor antagonist | 85 | 0.9955 |
| GABA receptor agonist | 912 | 0.9624 |
| GABA receptor antagonist | 3254 | 0.9892 |
| GABA uptake inhibitor | 69 | 0.9958 |
| Gastric antisecretory | 1683 | 0.9734 |
| Geranylgeranyltransferase inhibitor | 831 | 0.9947 |
| Geranyltranstransferase inhibitor | 204 | 0.9968 |
| GHS receptor agonist | 26 | 0.9888 |
| GHS receptor antagonist | 134 | 0.9975 |
| Glucagon receptor antagonist | 808 | 0.9956 |
| Glucagon-like peptide 1 agonist | 54 | 0.9812 |
| Glucagon-like peptide 1 receptor antagonist | 75 | 0.9997 |
| Glucocorticoid agonist | 901 | 0.9972 |
| Glucocorticoid antagonist | 1512 | 0.9955 |
| Glucose-6-phosphate dehydrogenase inhibitor | 44 | 0.995 |
| Glucose-6-phosphate isomerase inhibitor | 23 | 0.9992 |
| Glucose-6-phosphate translocase inhibitor | 45 | 0.9994 |
| Glucose-dependent insulinotropic receptor agonist | 670 | 0.9965 |
| Glucose-dependent insulinotropic receptor antagonist | 14 | 1 |
| Glucosylceramidase inhibitor | 296 | 0.9774 |
| Glutamate (mGluR group I) agonist | 675 | 0.9934 |
| Glutamate (mGluR group I) antagonist | 3286 | 0.9875 |
| Glutamate (mGluR group II) agonist | 375 | 0.9943 |
| Glutamate (mGluR group II) antagonist | 395 | 0.9983 |
| Glutamate (mGluR group III) agonist | 210 | 0.9919 |
| Glutamate (mGluR group III) antagonist | 157 | 0.9926 |
| Glutamate (mGluR) agonist | 1230 | 0.9896 |
| Glutamate (mGluR) antagonist | 3772 | 0.9879 |
| Glutamate (mGluR1) agonist | 114 | 0.9987 |
| Glutamate (mGluR1) antagonist | 1366 | 0.9918 |
| Glutamate (mGluR2) agonist | 366 | 0.9975 |
| Glutamate (mGluR2) antagonist | 296 | 0.9988 |
| Glutamate (mGluR3) agonist | 71 | 0.9965 |
| Glutamate (mGluR3) antagonist | 209 | 0.9947 |
| Glutamate (mGluR4) agonist | 188 | 0.9953 |
| Glutamate (mGluR4) antagonist | 67 | 0.9993 |
| Glutamate (mGluR5) agonist | 568 | 0.9926 |
| Glutamate (mGluR5) antagonist | 2106 | 0.9905 |
| Glutamate (mGluR6) agonist | 30 | 0.9997 |
| Glutamate (mGluR6) antagonist | 38 | 0.9834 |
| Glutamate (mGluR8) agonist | 26 | 0.9997 |
| Glutamate (mGluR8) antagonist | 46 | 0.9842 |
| Glutamate dehydrogenase inhibitor | 36 | 0.9699 |
| Glutamate receptor antagonist | 10595 | 0.9733 |
| Glutamate release inhibitor | 62 | 0.9611 |
| Glutathione S-transferase inhibitor | 116 | 0.9859 |
| Glycine receptor agonist | 15 | 0.9688 |
| Glycine receptor antagonist | 97 | 0.9792 |
| Glycine transporter 1 inhibitor | 940 | 0.9961 |
| Glycine transporter 2 inhibitor | 136 | 0.9885 |
| Glycine transporter inhibitor | 1046 | 0.9937 |
| Glycogen synthase kinase-3 inhibitor | 6589 | 0.973 |
| GP IIb/IIIa receptor antagonist | 2524 | 0.9923 |
| Granulocyte macrophage colony stimulating factor agonist | 12 | 0.9751 |
| Growth factor antagonist | 10451 | 0.9602 |
| Growth hormone agonist | 10 | 0.9734 |
| Growth hormone release inhibitor | 64 | 0.9979 |
| Growth hormone release promoting | 336 | 0.9976 |
| Growth hormone releasing factor agonist | 445 | 0.9961 |
| Growth hormone releasing factor antagonist | 41 | 0.9996 |
| Guanylate cyclase inhibitor | 32 | 0.9801 |
| Guanylate cyclase stimulant | 369 | 0.9801 |
| H+/K+-transporting ATPase inhibitor | 1053 | 0.9891 |
| HCV NS3/NS4A protease inhibitor | 1008 | 0.9869 |
| HCV NS5A inhibitor | 20 | 0.9996 |
| HCV NS5B polymerase inhibitor | 1080 | 0.9946 |
| HCV polyprotein inhibitor | 79 | 0.981 |
| Heat shock protein 70 antagonist | 23 | 0.965 |
| Heat shock protein 90 antagonist | 1342 | 0.9259 |
| Hematopoietic | 165 | 0.9653 |
| Heparanase inhibitor | 189 | 0.9944 |
| Hepatocyte growth factor antagonist | 2022 | 0.9682 |
| Hepatoprotectant | 545 | 0.9457 |
| Hexokinase inhibitor | 271 | 0.952 |
| Hexokinase stimulant | 637 | 0.9976 |
| Histamine agonist | 623 | 0.9835 |
| Histamine antagonist | 5351 | 0.9752 |
| Histamine H1 receptor agonist | 53 | 0.9857 |
| Histamine H1 receptor antagonist | 1710 | 0.9842 |
| Histamine H2 receptor agonist | 60 | 0.9742 |
| Histamine H2 receptor antagonist | 604 | 0.9846 |
| Histamine H3 receptor agonist | 313 | 0.9963 |
| Histamine H3 receptor antagonist | 3158 | 0.994 |
| Histamine H4 receptor agonist | 263 | 0.9981 |
| Histamine H4 receptor antagonist | 350 | 0.9962 |
| Histamine N-methyltransferase inhibitor | 73 | 0.9854 |
| Histidine decarboxylase inhibitor | 33 | 0.9845 |
| Histone deacetylase 1 inhibitor | 2264 | 0.9956 |
| Histone deacetylase 10 inhibitor | 395 | 0.9962 |
| Histone deacetylase 11 inhibitor | 359 | 0.9957 |
| Histone deacetylase 2 inhibitor | 778 | 0.9945 |
| Histone deacetylase 3 inhibitor | 668 | 0.9929 |
| Histone deacetylase 4 inhibitor | 609 | 0.995 |
| Histone deacetylase 5 inhibitor | 445 | 0.9914 |
| Histone deacetylase 6 inhibitor | 1127 | 0.9965 |
| Histone deacetylase 7 inhibitor | 436 | 0.9915 |
| Histone deacetylase 8 inhibitor | 600 | 0.9895 |
| Histone deacetylase 9 inhibitor | 400 | 0.9917 |
| Histone deacetylase class I inhibitor | 2597 | 0.9946 |
| Histone deacetylase class II inhibitor | 1263 | 0.996 |
| Histone deacetylase class III inhibitor | 69 | 0.9659 |
| Histone deacetylase class IV inhibitor | 359 | 0.9957 |
| Histone deacetylase inhibitor | 3792 | 0.9894 |
| Histone deacetylase SIRT1 inhibitor | 51 | 0.9624 |
| Histone deacetylase SIRT1 stimulant | 63 | 0.9926 |
| Histone deacetylase stimulant | 63 | 0.9926 |
| HIV-1 integrase (3'-Processing) inhibitor | 505 | 0.9861 |
| HIV-1 integrase (Overall Integration) inhibitor | 68 | 0.9845 |
| HIV-1 integrase (Strand Transfer) inhibitor | 527 | 0.9879 |
| HIV-1 integrase inhibitor | 1687 | 0.9837 |
| HIV-1 protease inhibitor | 5032 | 0.9935 |
| HIV-1 reverse transcriptase inhibitor | 2674 | 0.9883 |
| HMG CoA reductase inhibitor | 1366 | 0.9969 |
| Human herpes virus 5 capsid protein P40 inhibitor | 156 | 0.9997 |
| Human herpesvirus 2 protease inhibitor | 42 | 1 |
| Human T-cell leukemia virus type I protease inhibitor | 48 | 0.9996 |
| Hypoglycemic | 504 | 0.8992 |
| Hypolipemic | 9312 | 0.9397 |
| Hypoxanthine phosphoribosyltransferase inhibitor | 79 | 0.9964 |
| Hypoxia inducible factor 1 alpha inhibitor | 1716 | 0.8997 |
| I kappa B kinase inhibitor | 2275 | 0.9772 |
| Immunoglobulin Fc receptor antagonist | 86 | 0.9725 |
| Immunomodulator | 2776 | 0.9143 |
| Immunostimulant | 1135 | 0.929 |
| Immunosuppressant | 4252 | 0.8993 |
| Indoleamine-pyrrole 2,3-dioxygenase inhibitor | 230 | 0.9915 |
| Inducible nitric-oxide synthase inhibitor | 1206 | 0.982 |
| Inosine monophosphate dehydrogenase 1 inhibitor | 144 | 0.9952 |
| Inosine monophosphate dehydrogenase 2 inhibitor | 607 | 0.9994 |
| Insulin growth factor antagonist | 3012 | 0.9778 |
| Insulin like growth factor 1 antagonist | 2968 | 0.9776 |
| Insulin like growth factor 3 antagonist | 44 | 1 |
| Insulin receptor antagonist | 1005 | 0.9669 |
| Insulin secretagoues | 253 | 0.9651 |
| Insulin sensitizer | 237 | 0.9734 |
| Insulysin inhibitor | 71 | 0.8607 |
| Integrin alpha1beta1 antagonist | 42 | 0.9947 |
| Integrin alpha2 antagonist | 65 | 0.9922 |
| Integrin alpha2beta1 antagonist | 55 | 0.9938 |
| Integrin alpha4 antagonist | 2312 | 0.9977 |
| Integrin alpha4beta1 antagonist | 1819 | 0.9983 |
| Integrin alpha4beta7 antagonist | 809 | 0.9981 |
| Integrin alpha5beta1 antagonist | 237 | 0.9943 |
| Integrin alpha5beta6 antagonist | 14 | 1 |
| Integrin alphaLbeta2 antagonist | 38 | 0.9997 |
| Integrin alphaVbeta1 antagonist | 47 | 0.9968 |
| Integrin alphaVbeta3 antagonist | 781 | 0.9955 |
| Integrin alphaVbeta5 antagonist | 370 | 0.999 |
| Integrin alphaVbeta6 antagonist | 196 | 0.9995 |
| Integrin antagonist | 4598 | 0.9918 |
| Interferon agonist | 166 | 0.97 |
| Interferon gamma antagonist | 85 | 0.9552 |
| Interferon inducer | 144 | 0.9757 |
| Interleukin 1 antagonist | 922 | 0.9585 |
| Interleukin 1 beta converting enzyme inhibitor | 1416 | 0.9657 |
| Interleukin 1b antagonist | 500 | 0.969 |
| Interleukin 2 agonist | 17 | 0.9103 |
| Interleukin 2 antagonist | 244 | 0.9579 |
| Interleukin 4 antagonist | 147 | 0.953 |
| Interleukin 5 antagonist | 145 | 0.9703 |
| Interleukin 6 antagonist | 218 | 0.9352 |
| Interleukin 8 antagonist | 241 | 0.9512 |
| Interleukin agonist | 62 | 0.9236 |
| Interleukin antagonist | 1748 | 0.94 |
| Isoleucine-tRNA ligase inhibitor | 35 | 0.9985 |
| Janus tyrosine kinase 1 inhibitor | 1512 | 0.9944 |
| Janus tyrosine kinase 2 inhibitor | 4947 | 0.9796 |
| Janus tyrosine kinase 3 inhibitor | 4060 | 0.9865 |
| Kainate receptor 1 antagonist | 336 | 0.9984 |
| Kainate receptor 2 antagonist | 187 | 0.9966 |
| Kainate receptor 3 antagonist | 140 | 0.9988 |
| Kainate receptor 4 antagonist | 134 | 0.9986 |
| Kainate receptor 5 antagonist | 162 | 0.9986 |
| Kainate receptor agonist | 34 | 0.9873 |
| Kainate receptor antagonist | 565 | 0.9919 |
| Kallikrein inhibitor | 196 | 0.9652 |
| Keratolytic | 48 | 0.9652 |
| Kinesin antagonist | 1156 | 0.9944 |
| KiSS-1 receptor agonist | 35 | 1 |
| KiSS-1 receptor antagonist | 26 | 0.9995 |
| Lanosterol 14 alpha demethylase inhibitor | 95 | 0.9469 |
| Lck kinase inhibitor | 3408 | 0.9642 |
| Leukotriene A4 hydrolase inhibitor | 539 | 0.998 |
| Leukotriene antagonist | 2602 | 0.9857 |
| Leukotriene B4 antagonist | 913 | 0.988 |
| Leukotriene B4 receptor 1 antagonist | 572 | 0.9971 |
| Leukotriene D4 antagonist | 1041 | 0.996 |
| Leukotriene synthesis inhibitor | 819 | 0.9867 |
| LFA antagonist | 315 | 0.9974 |
| Lipase inhibitor | 237 | 0.9913 |
| Lipid peroxidase inhibitor | 735 | 0.9655 |
| Lipoxygenase inhibitor | 7641 | 0.9144 |
| Lysine carboxypeptidase inhibitor | 35 | 0.9983 |
| Macrophage elastase inhibitor | 426 | 0.9887 |
| Macrophage migration inhibitory factor inhibitor | 163 | 0.9789 |
| Maillard reaction inhibitor | 127 | 0.9907 |
| Mannitol 2-dehydrogenase inhibitor | 5 | 0.9999 |
| Mannitol-1-phosphate 5-dehydrogenase inhibitor | 18 | 0.9937 |
| Mannose-6-phosphate isomerase inhibitor | 126 | 0.9501 |
| Mannosidase inhibitor | 87 | 0.9909 |
| MAO A inhibitor | 1454 | 0.9821 |
| MAO B inhibitor | 1885 | 0.9867 |
| MAO inhibitor | 2771 | 0.9741 |
| MAP kinase 10 inhibitor | 1307 | 0.9794 |
| MAP kinase 11 inhibitor | 697 | 0.9757 |
| MAP kinase 14 inhibitor | 5881 | 0.9819 |
| MAP kinase 8 inhibitor | 1266 | 0.9688 |
| MAP kinase 9 inhibitor | 1005 | 0.9665 |
| MAP kinase kinase 1 inhibitor | 1160 | 0.9654 |
| MAP kinase kinase inhibitor | 1623 | 0.9685 |
| MAP kinase kinase kinase inhibitor | 1407 | 0.9765 |
| MAP-kinase-activated kinase inhibitor | 2166 | 0.9768 |
| MAP3K5 inhibitor | 94 | 0.9705 |
| MAP3K7 inhibitor | 98 | 0.987 |
| MAP3K8 inhibitor | 914 | 0.9953 |
| MAP3K9 inhibitor | 106 | 0.9508 |
| Matrix metalloproteinase (membrane-type) inhibitor | 1945 | 0.9875 |
| Matrix metalloproteinase 1 inhibitor | 2416 | 0.9902 |
| Matrix metalloproteinase inhibitor | 4037 | 0.9851 |
| MDM2 inhibitor | 506 | 0.9694 |
| Mediator release inhibitor | 469 | 0.9749 |
| Melanin inhibitor | 34 | 0.9555 |
| Melanin-concentrating hormone receptor 1 antagonist | 2484 | 0.9918 |
| Melanocortin 1 antagonist | 326 | 0.995 |
| Melanocortin 3 antagonist | 251 | 0.9984 |
| Melanocortin 4 antagonist | 828 | 0.9874 |
| Melanocortin 5 antagonist | 249 | 0.9988 |
| Melanocortin agonist | 1543 | 0.9974 |
| Melanocortin antagonist | 1006 | 0.9845 |
| Melatonin 1 agonist | 105 | 0.9991 |
| Melatonin 1 antagonist | 767 | 0.996 |
| Melatonin 2 agonist | 96 | 0.9986 |
| Melatonin 2 antagonist | 233 | 0.9989 |
| Melatonin 3 antagonist | 100 | 0.999 |
| Melatonin 5 antagonist | 68 | 0.9996 |
| Melatonin agonist | 315 | 0.9985 |
| Melatonin antagonist | 1008 | 0.9957 |
| Membrane dipeptidase inhibitor | 174 | 0.9894 |
| Metalloproteinase-2 inhibitor | 3026 | 0.9823 |
| Metalloproteinase-3 inhibitor | 1018 | 0.9924 |
| Metalloproteinase-9 inhibitor | 2465 | 0.9871 |
| Methylmalonyl-CoA mutase inhibitor | 11 | 0.994 |
| Microsomal triglyceride transfer protein inhibitor | 515 | 0.9992 |
| Microtubule formation inhibitor | 590 | 0.9618 |
| Microtubule stabilization | 511 | 0.9935 |
| Mineralocorticoid receptor antagonist | 664 | 0.9907 |
| Monophenol monooxygenase inhibitor | 287 | 0.9761 |
| Motilin receptor agonist | 319 | 0.9962 |
| Motilin receptor antagonist | 125 | 0.9993 |
| mTOR inhibitor | 5707 | 0.9901 |
| Mucolytic | 164 | 0.9644 |
| Myc inhibitor | 9 | 0.8919 |
| N-Acetylated alpha-linked acidic dipeptidase inhibitor | 261 | 0.9983 |
| Na+ K+ transporting ATPase inhibitor | 187 | 0.989 |
| NAD(P)H dehydrogenase (quinone) inhibitor | 174 | 0.9827 |
| NADH dehydrogenase inhibitor | 17 | 0.9594 |
| Nav1.1 sodium channel blocker | 167 | 0.9637 |
| Nav1.2 sodium channel blocker | 306 | 0.9658 |
| Nav1.3 sodium channel blocker | 168 | 0.9529 |
| Nav1.4 sodium channel blocker | 38 | 0.9528 |
| Nav1.5 sodium channel blocker | 278 | 0.9644 |
| Nav1.6 sodium channel blocker | 117 | 0.9585 |
| Nav1.7 sodium channel blocker | 1602 | 0.9918 |
| Nav1.8 sodium channel blocker | 126 | 0.9902 |
| Nerve growth factor antagonist | 40 | 0.9937 |
| Neuraminidase (Influenza A) inhibitor | 166 | 1 |
| Neuraminidase (Influenza B) inhibitor | 65 | 0.9999 |
| Neuraminidase (influenza) inhibitor | 196 | 0.9978 |
| Neuraminidase inhibitor | 420 | 0.9917 |
| Neurokinin 1 antagonist | 2486 | 0.9946 |
| Neurokinin 2 antagonist | 1527 | 0.9909 |
| Neurokinin 3 antagonist | 1255 | 0.9943 |
| Neurokinin antagonist | 3879 | 0.9914 |
| Neuronal nicotinic receptor antagonist | 1303 | 0.9866 |
| Neuropeptide agonist | 124 | 0.986 |
| Neuropeptide Y antagonist | 2041 | 0.9673 |
| Neuropeptide Y1 antagonist | 503 | 0.9392 |
| Neuropeptide Y2 antagonist | 223 | 0.9004 |
| Neuropeptide Y5 antagonist | 1043 | 0.9916 |
| Neurotensin receptor antagonist | 132 | 0.994 |
| Neurotrophic factor | 475 | 0.9627 |
| Neurotrophic factor enhancer | 73 | 0.9482 |
| Neutral endopeptidase inhibitor | 1401 | 0.9972 |
| Neutrophil collagenase inhibitor | 991 | 0.9887 |
| Nicotinic acid receptor agonist | 527 | 0.9942 |
| Nicotinic alpha4 receptor agonist | 93 | 0.9997 |
| Nicotinic alpha4beta2 receptor agonist | 218 | 0.9981 |
| Nicotinic alpha4beta2 receptor antagonist | 361 | 0.9841 |
| Nicotinic alpha7 receptor agonist | 809 | 0.9913 |
| Nicotinic neuronal receptor agonist | 1121 | 0.9913 |
| Nicotinic receptor alpha7 subunit antagonist | 253 | 0.9908 |
| Nitric oxide donor | 352 | 0.9927 |
| Nitric-oxide synthase inhibitor | 2135 | 0.9771 |
| Nitric-oxide synthase stimulant | 19 | 0.9083 |
| NMDA 2 receptor antagonist | 941 | 0.9928 |
| NMDA 2A receptor antagonist | 568 | 0.9967 |
| NMDA 2B receptor antagonist | 396 | 0.992 |
| NMDA receptor agonist | 181 | 0.9843 |
| NMDA receptor antagonist | 5037 | 0.9832 |
| NOS3 expression enhancer | 100 | 0.9492 |
| Opioid agonist | 3116 | 0.9907 |
| Opioid antagonist | 4814 | 0.9723 |
| Opioid delta receptor agonist | 1302 | 0.9935 |
| Opioid delta receptor antagonist | 2699 | 0.9893 |
| Opioid kappa receptor agonist | 1178 | 0.9906 |
| Opioid kappa receptor antagonist | 2500 | 0.9702 |
| Opioid mu receptor agonist | 1133 | 0.9909 |
| Opioid mu receptor antagonist | 3394 | 0.9862 |
| Orexin receptor antagonist | 1421 | 0.9625 |
| Ornithine carbamoyltransferase inhibitor | 40 | 0.9935 |
| Ornithine decarboxylase inhibitor | 257 | 0.9877 |
| Oxytocin agonist | 39 | 0.9588 |
| Oxytocin antagonist | 1164 | 0.9922 |
| p53 inhibitor | 29 | 0.9434 |
| Parathyroid hormone antagonist | 23 | 0.9689 |
| Peptidyl-prolyl cis-trans isomerase inhibitor | 21997 | 0.8893 |
| Peptidyltransferase inhibitor | 20 | 0.9135 |
| Peroxidase inhibitor | 16 | 0.9433 |
| Peroxisome proliferator-activated receptor alpha agonist | 2281 | 0.9948 |
| Peroxisome proliferator-activated receptor alpha antagonist | 559 | 0.9973 |
| Peroxisome proliferator-activated receptor delta agonist | 1287 | 0.9973 |
| Peroxisome proliferator-activated receptor delta antagonist | 299 | 0.9931 |
| Peroxisome proliferator-activated receptor gamma agonist | 2560 | 0.9895 |
| Peroxisome proliferator-activated receptor gamma antagonist | 1021 | 0.9736 |
| Phenylalanine 4-hydroxylase inhibitor | 34 | 0.9911 |
| Phosphatidylinositol kinase inhibitor | 9223 | 0.9887 |
| Phosphodiesterase I inhibitor | 527 | 0.9704 |
| Phosphodiesterase II inhibitor | 319 | 0.9885 |
| Phosphodiesterase III inhibitor | 1434 | 0.9881 |
| Phosphodiesterase inhibitor | 10558 | 0.9682 |
| Phosphodiesterase IV inhibitor | 5372 | 0.985 |
| Phosphodiesterase IX inhibitor | 64 | 0.9992 |
| Phosphodiesterase V inhibitor | 2227 | 0.9856 |
| Phosphodiesterase VI inhibitor | 278 | 0.9939 |
| Phosphodiesterase VII inhibitor | 502 | 0.9878 |
| Phosphodiesterase VIII inhibitor | 124 | 0.991 |
| Phosphodiesterase X inhibitor | 994 | 0.9939 |
| Phosphodiesterase XI inhibitor | 103 | 0.9867 |
| Phosphofructokinase-1 inhibitor | 33 | 0.9902 |
| Phosphoglycerate kinase inhibitor | 189 | 0.9969 |
| Phospholipase A2 inhibitor | 1606 | 0.9467 |
| Phospholipase C inhibitor | 81 | 0.9602 |
| Phospholipase D inhibitor | 125 | 0.9763 |
| Phosphoribosylglycinamide formyltransferase inhibitor | 198 | 0.998 |
| Phosphorylase inhibitor | 602 | 0.9913 |
| Photosensitizer | 76 | 0.9537 |
| Plasmin inhibitor | 590 | 0.9895 |
| Plasminogen activator inhibitor | 201 | 0.9881 |
| Plasminogen activator inhibitor antagonist | 309 | 0.994 |
| Platelet activating factor alpha antagonist | 1781 | 0.9757 |
| Platelet activating factor antagonist | 6895 | 0.9716 |
| Platelet activating factor beta antagonist | 3422 | 0.9775 |
| Platelet aggregation inhibitor | 11688 | 0.941 |
| Platelet antagonist | 2010 | 0.9577 |
| Platelet growth factor antagonist | 933 | 0.9821 |
| Polo-like kinase-1 inhibitor | 1280 | 0.9734 |
| Polo-like kinase-2 inhibitor | 141 | 0.9704 |
| Polo-like kinase-3 inhibitor | 318 | 0.9416 |
| Polo-like kinase-4 inhibitor | 697 | 0.9677 |
| Poly(ADP-ribose) polymerase inhibitor | 2188 | 0.99 |
| Porphobilinogen synthase inhibitor | 22 | 0.9891 |
| Potassium channel (ATP-sensitive) activator | 496 | 0.9894 |
| Potassium channel (ATP-sensitive) blocker | 635 | 0.986 |
| Potassium channel (Ca-activated) activator | 250 | 0.9719 |
| Potassium channel (Ca-activated) blocker | 322 | 0.9802 |
| Potassium channel (Inward rectifier) activator | 496 | 0.9894 |
| Potassium channel (Inward rectifier) blocker | 831 | 0.9881 |
| Potassium channel (Voltage-sensitive) activator | 343 | 0.9582 |
| Potassium channel (Voltage-sensitive) blocker | 5628 | 0.9435 |
| Potassium channel activator | 2527 | 0.9749 |
| Potassium channel blocker | 9159 | 0.9445 |
| Potassium channel intermediate-conductance Ca-activated blocker | 93 | 0.9896 |
| Potassium channel Kv1.1 blocker | 138 | 0.9824 |
| Potassium channel Kv1.3 blocker | 419 | 0.9919 |
| Potassium channel Kv1.5 blocker | 852 | 0.9861 |
| Potassium channel small-conductance Ca-activated blocker | 159 | 0.9895 |
| Progesterone agonist | 534 | 0.9989 |
| Progesterone antagonist | 1491 | 0.9957 |
| Progesterone receptor A antagonist | 98 | 0.9999 |
| Progesterone receptor B antagonist | 66 | 0.9994 |
| Prokineticin receptor 1 antagonist | 429 | 1 |
| Prolactin inhibitor | 44 | 0.9777 |
| Prolactin release inhibitor | 65 | 0.9734 |
| Prolyl endopeptidase inhibitor | 764 | 0.9921 |
| Prostacyclin agonist | 103 | 0.9913 |
| Prostacyclin antagonist | 296 | 0.9961 |
| Prostaglandin agonist | 1439 | 0.9944 |
| Prostaglandin antagonist | 4791 | 0.9883 |
| Prostaglandin D2 agonist | 80 | 0.9975 |
| Prostaglandin D2 antagonist | 1945 | 0.9983 |
| Prostaglandin E2 agonist | 1125 | 0.9974 |
| Prostaglandin E2 antagonist | 2408 | 0.991 |
| Prostaglandin EP2 agonist | 81 | 0.995 |
| Prostaglandin EP2 antagonist | 224 | 0.9963 |
| Prostaglandin EP3 antagonist | 583 | 0.998 |
| Prostaglandin EP4 agonist | 167 | 0.9996 |
| Prostaglandin EP4 antagonist | 339 | 0.9939 |
| Prostaglandin F2 alpha agonist | 89 | 0.9879 |
| Prostaglandin F2 alpha antagonist | 60 | 0.9665 |
| Protease activated receptor 1 agonist | 59 | 0.9999 |
| Protease activated receptor 1 antagonist | 577 | 0.9946 |
| Protease activated receptor agonist | 100 | 0.9998 |
| Protease activated receptor antagonist | 600 | 0.9901 |
| Protease inhibitor | 1101 | 0.9654 |
| Protein 30S ribosomal subunit inhibitor | 68 | 0.9751 |
| Protein 50S ribosomal subunit inhibitor | 112 | 0.9911 |
| Protein kinase (CaMK, MLCK, PhK, SNF, KIN, NIM1, MAPKAP, POLO, CHK, ULK, RSK-2nd domain) inhibitor | 3417 | 0.9801 |
| Protein kinase (CK1) inhibitor | 970 | 0.9594 |
| Protein kinase (CK2) inhibitor | 918 | 0.971 |
| Protein kinase (Mos, Mil/Raf, MEKK, RIPK, TESK, LIMK, IRAK, ILK, Activin/TGF-beta) inhibitor | 4795 | 0.9777 |
| Protein kinase A inhibitor | 1728 | 0.9788 |
| Protein kinase B alpha inhibitor | 1096 | 0.988 |
| Protein kinase B beta inhibitor | 673 | 0.9728 |
| Protein kinase B gamma inhibitor | 669 | 0.9766 |
| Protein kinase C alpha inhibitor | 1729 | 0.9843 |
| Protein kinase C beta I inhibitor | 284 | 0.9919 |
| Protein kinase C beta II inhibitor | 535 | 0.9965 |
| Protein kinase C delta inhibitor | 1381 | 0.9751 |
| Protein kinase C epsilon inhibitor | 1154 | 0.9895 |
| Protein kinase C eta inhibitor | 544 | 0.986 |
| Protein kinase C gamma inhibitor | 776 | 0.9683 |
| Protein kinase C iota inhibitor | 222 | 0.9711 |
| Protein kinase C mu inhibitor | 270 | 0.9769 |
| Protein kinase C theta inhibitor | 2853 | 0.9884 |
| Protein kinase C zeta inhibitor | 314 | 0.9728 |
| Protein kinase stimulant | 128 | 0.9641 |
| Protein-tyrosine kinase (PTK, not ETK, WZC) inhibitor | 2513 | 0.9677 |
| Protein-tyrosine phosphatase inhibitor | 2955 | 0.9549 |
| Proto-oncogene tyrosine-protein kinase c-hck inhibitor | 346 | 0.9837 |
| Proto-oncogene tyrosine-protein kinase Fyn inhibitor | 768 | 0.9283 |
| Proto-oncogene tyrosine-protein kinase Kit inhibitor | 2549 | 0.9814 |
| Proto-oncogene tyrosine-protein kinase Met inhibitor | 4740 | 0.9924 |
| Proto-oncogene tyrosine-protein kinase Yes inhibitor | 163 | 0.9534 |
| Protocollagen prolyl hydroxylase inhibitor | 406 | 0.996 |
| Psychostimulant | 417 | 0.9432 |
| Purine nucleoside phosphorylase inhibitor | 379 | 0.9981 |
| Purinergic P2T antagonist | 231 | 0.9982 |
| Purinergic P2X antagonist | 2934 | 0.9895 |
| Purinergic P2Y antagonist | 1100 | 0.9935 |
| Purinergic receptor agonist | 379 | 0.9987 |
| Pyruvate kinase inhibitor | 492 | 0.9374 |
| Raf kinase inhibitor | 4289 | 0.9858 |
| Renin inhibitor | 3992 | 0.9955 |
| RET inhibitor | 805 | 0.9602 |
| Retinoic acid alpha receptor agonist | 75 | 0.9993 |
| Retinoic acid alpha receptor antagonist | 256 | 0.9984 |
| Retinoic acid beta receptor agonist | 101 | 0.9996 |
| Retinoic acid beta receptor antagonist | 257 | 0.9994 |
| Retinoic acid gamma receptor agonist | 62 | 0.9996 |
| Retinoic acid receptor agonist | 257 | 0.9967 |
| Retinoic acid receptor antagonist | 401 | 0.9975 |
| Retinoic acid receptor gamma antagonist | 290 | 0.9967 |
| Retinoid X receptor agonist | 242 | 0.995 |
| Retinoid X receptor antagonist | 281 | 0.9969 |
| Reverse transcriptase (Hepatitis B) inhibitor | 36 | 1 |
| Rho-associated kinase I inhibitor | 2696 | 0.9827 |
| Rho-associated kinase inhibitor | 4232 | 0.9814 |
| Ribonucleoside diphosphate reductase inhibitor | 155 | 0.9872 |
| Ribonucleotide reductase inhibitor | 288 | 0.991 |
| Ribosomal protein S6 kinase inhibitor | 1691 | 0.9561 |
| RNA-directed RNA polymerase inhibitor | 26 | 0.8816 |
| RNA-directed RNA polymerase stimulant | 7 | 0.9992 |
| Ryanodine receptor antagonist | 8 | 1 |
| S-adenosyl-L-homocysteine hydrolase inhibitor | 167 | 0.9997 |
| S-adenosyl-L-methionine decarboxylase inhibitor | 58 | 0.9989 |
| Secretase beta inhibitor | 2789 | 0.9863 |
| Secretase gamma inhibitor | 418 | 0.9974 |
| Selectin antagonist | 301 | 0.9854 |
| Sigma receptor agonist | 23 | 0.9475 |
| Sigma receptor antagonist | 2239 | 0.9907 |
| Smo receptor antagonist | 374 | 0.9953 |
| Sodium channel blocker | 5445 | 0.9657 |
| Somatostatin 1 antagonist | 240 | 0.9955 |
| Somatostatin 2 antagonist | 361 | 0.994 |
| Somatostatin 3 antagonist | 319 | 0.9943 |
| Somatostatin 4 antagonist | 323 | 0.9935 |
| Somatostatin 5 antagonist | 331 | 0.9917 |
| Somatostatin agonist | 91 | 0.9947 |
| Spasmolytic | 1668 | 0.9222 |
| Sphingosine 1-phosphate receptor 1 agonist | 1392 | 0.9927 |
| Sphingosine 1-phosphate receptor 1 antagonist | 498 | 0.969 |
| Sphingosine 1-phosphate receptor 3 agonist | 410 | 0.9973 |
| Sphingosine 1-phosphate receptor 3 antagonist | 174 | 0.9754 |
| Sphingosine 1-phosphate receptor 5 agonist | 90 | 0.9905 |
| Sphingosine 1-phosphate receptor 5 antagonist | 96 | 0.9821 |
| Sphingosine 1-phosphate receptor agonist | 2270 | 0.989 |
| Sphingosine 1-phosphate receptor antagonist | 734 | 0.9574 |
| Sphingosine kinase inhibitor | 160 | 0.9969 |
| Squalene epoxidase inhibitor | 191 | 0.9929 |
| Squalene synthetase inhibitor | 1104 | 0.9959 |
| Src kinase inhibitor | 8018 | 0.9583 |
| Steroid 17-alpha-hydroxylase/17,20 lyase inhibitor | 596 | 0.9958 |
| Substance P antagonist | 1648 | 0.9929 |
| Succinate dehydrogenase inhibitor | 14 | 0.9606 |
| Superoxide dismutase inhibitor | 16 | 0.9067 |
| Syk tyrosine kinase inhibitor | 1401 | 0.976 |
| Telomerase inhibitor | 685 | 0.9732 |
| Telomerase stimulant | 6 | 1 |
| Thiazolidinedione | 26 | 0.9964 |
| Thiol protease inhibitor | 413 | 0.9287 |
| Thioredoxin reductase inhibitor | 90 | 0.945 |
| Thrombin inhibitor | 5032 | 0.977 |
| Thrombolytic | 392 | 0.9757 |
| Thrombopoietin agonist | 271 | 0.9998 |
| Thromboxane agonist | 49 | 0.9997 |
| Thromboxane antagonist | 1445 | 0.9882 |
| Thromboxane synthase inhibitor | 1531 | 0.9845 |
| Thymidine kinase (Herpes simplex virus 1) inhibitor | 139 | 0.9988 |
| Thymidine kinase (Herpes simplex virus 2) inhibitor | 124 | 0.9999 |
| Thymidine kinase inhibitor | 313 | 0.9898 |
| Thymidine phosphorylase inhibitor | 106 | 0.9931 |
| Thymidylate synthase inhibitor | 1481 | 0.9931 |
| Thyroid hormone agonist | 165 | 0.9924 |
| Thyroid hormone alpha agonist | 39 | 0.9999 |
| Thyroid hormone alpha antagonist | 183 | 0.9982 |
| Thyroid hormone beta agonist | 111 | 0.9998 |
| TIE antagonist | 1866 | 0.9799 |
| Toll-Like receptor agonist | 9136 | 0.8982 |
| Toll-Like receptor antagonist | 240 | 0.951 |
| Topoisomerase I inhibitor | 874 | 0.9775 |
| Topoisomerase II inhibitor | 1119 | 0.9749 |
| Transcortin receptor antagonist | 29 | 0.9998 |
| Transcription factor AP-1 inhibitor | 306 | 0.9726 |
| Transcription factor inhibitor | 22815 | 0.8517 |
| Transcription factor NF kappa B inhibitor | 1009 | 0.9102 |
| Transcription factor NF kappa B stimulant | 7 | 0.8819 |
| Transcription factor RelA inhibitor | 72 | 0.9656 |
| Transcription factor STAT inhibitor | 491 | 0.8689 |
| Transcription factor STAT3 inhibitor | 362 | 0.8855 |
| Transforming growth factor antagonist | 1046 | 0.9912 |
| Triose-phosphate isomerase inhibitor | 23 | 0.9909 |
| TRPA1 agonist | 144 | 0.9843 |
| TRPA1 antagonist | 201 | 0.9918 |
| Trypsin I inhibitor | 973 | 0.9941 |
| Trypsin II inhibitor | 188 | 0.997 |
| Trypsin inhibitor | 2067 | 0.972 |
| Tryptase inhibitor | 451 | 0.9928 |
| Tubulin agonist | 33 | 0.9964 |
| Tubulin antagonist | 1515 | 0.9759 |
| Tumour necrosis factor alpha release inhibitor | 2463 | 0.9478 |
| Tumour necrosis factor antagonist | 948 | 0.929 |
| Tyrosine 3 hydroxylase inhibitor | 12 | 0.8985 |
| Tyrosine kinase inhibitor | 27061 | 0.9468 |
| Tyrosine-protein kinase EMT inhibitor | 1729 | 0.9891 |
| Tyrosine-protein kinase receptor antagonist | 1162 | 0.9596 |
| Tyrosine-protein kinase receptor FLT3 inhibitor | 2796 | 0.9744 |
| UDP-glucose 4-epimerase inhibitor | 28 | 0.9762 |
| UDP-N-acetylglucosamine 1-carboxyvinyltransferase inhibitor | 22 | 0.9619 |
| Urease inhibitor | 42 | 0.978 |
| Uric acid excretion stimulant | 250 | 0.9622 |
| Uridine phosphorylase inhibitor | 125 | 0.9985 |
| Urokinase inhibitor | 845 | 0.9939 |
| Urotensin II agonist | 81 | 0.9976 |
| Urotensin II antagonist | 455 | 0.9982 |
| Vanilloid agonist | 183 | 0.9776 |
| Vanilloid antagonist | 2251 | 0.9875 |
| Vascular endothelial growth factor 1 antagonist | 1884 | 0.9705 |
| Vascular endothelial growth factor 2 antagonist | 12039 | 0.973 |
| Vascular endothelial growth factor 3 antagonist | 1107 | 0.9607 |
| Vascular endothelial growth factor antagonist | 12888 | 0.9678 |
| Vasodilator | 1692 | 0.9254 |
| Vasodilator, coronary | 288 | 0.8844 |
| Vasodilator, peripheral | 225 | 0.8816 |
| Vasopressin 1 agonist | 80 | 0.9814 |
| Vasopressin 1 antagonist | 1355 | 0.996 |
| Vasopressin 2 agonist | 212 | 0.9985 |
| Vasopressin 2 antagonist | 876 | 0.9957 |
| VCAM antagonist | 438 | 0.9896 |
| VCAM-1 antagonist | 437 | 0.9896 |
| Vitamin D receptor agonist | 102 | 0.9997 |
| Vitamin D receptor antagonist | 163 | 0.9901 |
| VLA-4 antagonist | 1121 | 0.9969 |
| Xanthine dehydrogenase inhibitor | 275 | 0.9836 |
| Xanthine oxidase inhibitor | 231 | 0.9756 |
