# Supplementary material for: Computer-Aided Estimation of Biological Activity Profiles of Drug-Like Compounds Taking into Account Their Metabolism in Human Body
Source: Int J Mol Sci. 2020 Oct 11;21(20):7492. doi: 10.3390/ijms21207492 (PMC7593915; doi:10.3390/ijms21207492)
Supplement: Supplementary file 1 [file ijms-21-07492-s001.zip › Filimonov_DA-et-al-Table_S4.docx]

**Table S4.** Lists of biological activities belonging to the category “Toxic and Adverse Effects”. NA is the number of active compounds; IAP is an Invariant Accuracy of Prediction obtained in leave-one-out cross-validation.

| **Activity** | **NA** | **IAP, LOO CV** |
| --- | --- | --- |
| Abortion inducer | 42 | 0.9607 |
| Carcinogenic | 2474 | 0.9303 |
| Carcinogenic, group 1 | 23 | 0.8776 |
| Carcinogenic, group 2A | 36 | 0.9636 |
| Carcinogenic, group 2B | 194 | 0.9683 |
| Carcinogenic, group 3 | 386 | 0.9585 |
| Carcinogenic, mouse | 456 | 0.9320 |
| Carcinogenic, mouse, female | 384 | 0.9267 |
| Carcinogenic, mouse, male | 365 | 0.9409 |
| Carcinogenic, rat | 602 | 0.9429 |
| Carcinogenic, rat, female | 444 | 0.9383 |
| Carcinogenic, rat, male | 510 | 0.9419 |
| Cardiodepressant | 165 | 0.9369 |
| Cytotoxic | 956 | 0.9396 |
| Embryotoxic | 1755 | 0.9231 |
| Eye irritation, high | 528 | 0.9480 |
| Genotoxic | 184 | 0.9461 |
| Lacrimal secretion stimulant | 85 | 0.9149 |
| Mutagenic | 5583 | 0.9429 |
| Pneumotoxic | 23 | 0.9255 |
| Sedative | 1530 | 0.9369 |
| Sensitization | 183 | 0.9387 |
| Skin irritative effect | 1140 | 0.9645 |
| Teratogen | 1561 | 0.9229 |
| Toxic, respiratory center | 15 | 0.9366 |
| Ulceration | 65 | 0.8621 |
| DNA damaging | 476 | 0.9700 |
| Hypoglycemic | 504 | 0.8992 |
| Abortion inducer | 42 | 0.9607 |
| Carcinogenic | 2474 | 0.9303 |
| Carcinogenic, group 1 | 23 | 0.8776 |
| Carcinogenic, group 2A | 36 | 0.9636 |
| Carcinogenic, group 2B | 194 | 0.9683 |
| Carcinogenic, group 3 | 386 | 0.9585 |
| Carcinogenic, mouse | 456 | 0.9320 |
| Carcinogenic, mouse, female | 384 | 0.9267 |
| Carcinogenic, mouse, male | 365 | 0.9409 |
| Carcinogenic, rat | 602 | 0.9429 |
| Carcinogenic, rat, female | 444 | 0.9383 |
| Carcinogenic, rat, male | 510 | 0.9419 |
| Cardiodepressant | 165 | 0.9369 |
| Cytotoxic | 956 | 0.9396 |
| Embryotoxic | 1755 | 0.9231 |
| Eye irritation, high | 528 | 0.9480 |
| Genotoxic | 184 | 0.9461 |
| Lacrimal secretion stimulant | 85 | 0.9149 |
| Mutagenic | 5583 | 0.9429 |
| Pneumotoxic | 23 | 0.9255 |
| Sedative | 1530 | 0.9369 |
| Sensitization | 183 | 0.9387 |
| Skin irritative effect | 1140 | 0.9645 |
| Teratogen | 1561 | 0.9229 |
| Toxic, respiratory center | 15 | 0.9366 |
| Ulceration | 65 | 0.8621 |
| DNA damaging | 476 | 0.9700 |
| Hypoglycemic | 504 | 0.8992 |
